# Polymeric Self-Assemblies Based on tetra-*ortho*-Substituted Azobenzene as Visible Light Responsive Nanocarriers

**DOI:** 10.3390/polym11122060

**Published:** 2019-12-11

**Authors:** Alejandro Roche, Luis Oriol, Rosa M. Tejedor, Milagros Piñol

**Affiliations:** 1 Departamento de Química Orgánica, Instituto de Ciencia de Materiales de Aragón (ICMA), Universidad de Zaragoza‒CSIC, c/Pedro Cerbuna 12, 50009 Zaragoza, Spain; roche@unizar.es (A.R.); loriol@unizar.es (L.O.); 2 Centro Universitario de la Defensa, Academia General Militar, Ctra. de Huesca s/n, 50090 Zaragoza, Spain

**Keywords:** azobenzene, block copolymers, self-assembly, nanocarriers, light response

## Abstract

Most of reported polymeric light-responsive nanocarriers make use of UV light to trigger morphological changes and the subsequent release of encapsulated cargoes. Moving from UV- to visible-responsive units is interesting for the potential biomedical applications of these materials. Herein we report the synthesis by ring opening polymerization (ROP) of a series of amphiphilic diblock copolymers, into which either UV or visible responsive azobenzenes have been introduced via copper(I) catalyzed azide-alkyne cycloaddition (CuAAC). These copolymers are able to self-assemble into spherical micelles or vesicles when dispersed in water. The study of the response of the self-assemblies upon UV (365 nm) or visible (530 or 625 nm) light irradiation has been studied by Transmission Electron Microscopy (TEM), Cryogenic Transmission Electron Microscopy (Cryo-TEM), and Dynamic Light Scattering (DLS) studies. Encapsulation of Nile Red, in micelles and vesicles, and Rhodamine B, in vesicles, and its light-stimulated release has been studied by fluorescence spectroscopy and confocal microscopy. Appreciable morphological changes have been induced with green light, and the subsequent release of encapsulated cargoes upon green light irradiation has been confirmed.

## 1. Introduction

Over the last years, polymeric micelles and vesicles formed through the self-assembly of amphiphilic copolymers have deserved great interest as nanocarriers of therapeutic agents since their physicochemical properties and performance can be adjusted by fine-tuning of the polymers chemical structure [1,2]. When dispersed in water, amphiphilic diblock copolymers (BCs) consisting of a hydrophilic and a hydrophobic segment bound together are able to self-assemble into nanometric objects whose morphology relies on factors such as the polymer topology, the length of each block, or the hydrophilic/hydrophobic balance [3,4,5]. In polymeric spherical micelles, the hydrophobic segments interact forming a core that can behave as a basin for transporting hydrophobic small molecules across aqueous media, while the hydrophilic segments are exposed to these aqueous media to stabilize the micellar structure [1,6]. In polymeric vesicles, which are larger in size, the hydrophilic chains assemble forming two coronas that face the internal aqueous cavity and the surrounding aqueous medium, with the hydrophobic chain separating them. These vesicular self-assemblies can be used for the encapsulation and transport of both hydrophilic and hydrophobic small molecules inside the hollow cavity or the bilayer, respectively. This encapsulation ability of micelles and vesicles can be exploited to fabricate suitable drug delivery vehicles in medical treatments to reduce classical limitations of conventional medical therapies such as toxicity, poor selectivity, or low solubility of hydrophobic drugs [7,8].

On demand release of any encapsulated payload can be attained if incorporating stimuli-sensitive moieties into the structure of the BCs. The application of a stimulus may cause the alteration of the polymer self-assemblies triggering the release of the payloads. Amongst all, light is an especially valuable stimulus as it can be easily tuned and spatially and temporally controlled [9]. Azobenzene has been probably the most widely used light-responsive moiety as it can undergo a photoinduced reversible *trans*-*cis* isomerization accompanied by a change in the geometry and polarity from which the light-induced release originates. There are numerous examples of amphiphilic BCs comprising azobenzene moieties that have been used for the fabrication light-responsive self-assemblies working under the application of UV light [10]. This is an evident disadvantage when biological applications are observed since UV light can induce harmful processes into cells, including apoptosis, besides having a low penetration in biological tissues [11]. There are several examples in literature on azobenzenes whose absorption is displaced to the visible region of the electromagnetic spectrum, but usually shifting azobenzene absorption to longer wavelengths implies faster thermal relaxation states and could led to change in the energies of the *trans* and *cis* states that disrupt the photoswitching properties [12]. BF_2_-adducts of azobenzenes reported by Aprahamanian group have the π−π* transitions centered in the visible region and have good photoswitching properties but are not useful for biological applications as they are degraded to hydrazones in water [13,14]. Two-photon absorption azobenzenes via direct excitation or via antenna effect have been reported but usually those molecules are too wide and two-photon absorption requires high energy femtoseconds lasers [15]. Woolley and co-workers have reported a series of tetra-*ortho-*substituted azobenzene derivatives with separated *E* and *Z* n−π* absorption bands, having a photoresponse to visible light [16,17]. Wang and co-workers reported an amphiphilic random copolymer formed by hydrophobic acrylate units functionalized with a tetra-*ortho-*substituted azobenzene and hydrophilic acrylic acid units that self-assembled into spherical micelles that were able to load small molecules as Nile Red, though the release of encapsulated cargoes upon visible light irradiation was not definitely established [18].

**Figure 1:**
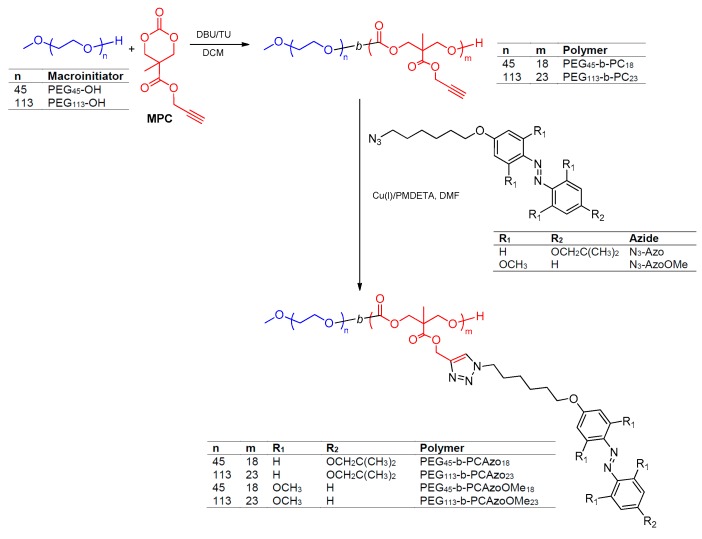
Route for the synthesis of the azobenzene functionalized amphiphilic block copolymers.

In early reports, we described a series of light responsive amphiphilic linear-dendritic diblock copolymers consisting of a linear hydrophilic segment of poly (ethylene glycol) (PEG) linked to a 2,2-di(hydroxymethyl)propionic acid (bis-MPA) based dendron as the hydrophobic block whose periphery was decorated with azobenzenes [19,20]. When coupling the bis-MPA dendron of the 4th generation bearing sixteen 4-isobutyloxyazobenzenes to a PEG segment with an average molecular mass of 2 kDa, vesicles were formed through the self-assembly process. 4-Isobutyloxyazobenzene promoted the light-triggered response so that vesicles were able to encapsulate Nile Red and Rhodamine B fluorescent probes and release them by exposing them to a low intensity UV lamp. The unresolved limitation of these vesicles was the need of UV light to induce the release. Persuaded by the possibility of using more suitable light wavelengths, we present here a series linear-linear amphiphilic diblock copolymers based on 2,2′,5,5′-tetramethoxyazobenzene unit to explore the feasibility of producing polymeric self-assemblies with release abilities under visible light stimulation (Figure 1). Likewise, these linear-linear BCs have been designed consisting of PEG as the hydrophilic segment bind to a hydrophobic aliphatic polycarbonate based on the bis-MPA building block. The presence of two hydroxyl and one carboxylic groups makes bis-MPA a remarkably versatile basic unit from which diverse polymeric architectures have been prepared ranging from dendrons and dendrimers to linear polymers either with bis-MPA based pending units or forming the polymer main chain [21,22]. Additionally, it is usually anticipated that the presence of ester or carbonate bonds hydrolysable bonds on the resulting polymers potentially confers biodegradability, which makes them valuable candidates for biomedical applications [23,24,25]. In order to gain direct comparisons, the linear-linear version with 4-isobutyloxyazobenzene (used as a proof-of-concept in earlier works) is also presented.

## 2. Materials and Methods 

### 2.1. General Synthetic Procedures

The cyclic carbonate 5-methyl-5-propargyloxycarbonyl-1,3-dioxan-2-one (MPC) was synthesized according to a reported procedure [26]. Details of the synthesis and characterization of the azides N_3_-Azo y N_3_-AzoOMe are described at the Supporting Information. Poly(ethylene glycol) methyl ethers PEG_113_–OH and PEG_45_–OH were purchased from Sigma Aldrich (Sigma Aldrich Gmbh, Steinheim, Germany) and used as received (for PEG_113_–OH *M*_n_ = 4431 g mol^−1^, *Ð* = 1.28 according to certificate of analysis, lot 1337809; for PEG_45_–OH *M*_n_ = 1678 g mol^−1^, *Ð* = 1.21 according to certificate of analysis, lot 1257344). 1-(3,5-Bis(trifluoromethyl)phenyl)-3-cyclohexylthiourea (TU) was synthesized as described in the literature [27]. 1,8-Diazabicyclo(5.4.0)undec-7-ene (DBU) was purchased from Alfa Aesar (Alfa Aesar, Heysham, UK) and used as received. *N,N,N′,N″,N″*-Pentamethyldiethylenetriamine (PMDETA) was purchased from Sigma Aldrich and used as received.

#### 2.1.1. General Procedure for the Synthesis of PEG_n_-*b*-PC_n_ Block Copolymers

A solution of PEG_n_–OH (1 mol), DBU (1% mol to monomer), and TU (5% mol to monomer) in dry dichloromethane ([MPC]_0_ = 1.0 M) was previously dried for 12 h over activated 4 Å molecular sieves under Ar atmosphere. This solution was added via cannula to a Schlenk flask charged with MPC (23 mol for PEG_45_–OH, 30 mol for PEG_113_–OH) under Ar atmosphere. The reaction was stirred at 35 °C for 8 h, then concentrated under vacuum. PEG_113_-*b*-PC_23_ was precipitated in cold diethyl ether and isolated as a white power by vacuum filtration. PEG_45_-*b*-PC_18_, which was soluble in cold diethyl ether, was isolated by silica column chromatography using dichloromethane/ethyl acetate (8/2) as eluent. e.g., PEG_113_-*b*-PC_23_: FTIR (KBr disk. cm^−1^): 3471 (O–H), 3290 (Csp–H), 2887 (Csp^3^–H), 2130 (Csp–Csp), 1754 (C=O). ^1^H NMR (400 MHz, CDCl_3_, δ, ppm]: 4.66 (d, *J* = 2.4 Hz), 4.34–4.15 (m), 3.79–3.36 (m), 3.31 (s), 2.48 (*t*, *J* = 2.4 Hz), 1.22 (s). GPC data: PEG_113_-*b*-PC_23_ *M*_n_ = 9460, *Ð* = 1.06; PEG_45_-*b*-PC_18_ *M*_n_ = 6810, *Ð* = 1.08.

#### 2.1.2. General Procedure for the Side Chain Functionalization by CuAAC

A Schlenk flask charged with the azide N_3_-Azo or N_3_-AzoOMe (2 mol), the propargyl functionalized copolymer PEG_n_-*b*-PC_m_ (1 mol of propargyl group), CuBr (0.3 mol) and PMDETA (0.3 mol) was flushed with Ar. Then, deoxygenated and distilled DMF (5 mL) was added. The reaction was maintained at 40 °C for 5 days. The crude reaction was diluted with dichloromethane and washed three times with distilled water. The organic fraction was dried over MgSO_4_ and evaporated to dryness. Residual azide was removed by preparative SEC using Biobeads^TM^ SX-1 and dichloromethane as eluent. Copolymer containing fractions were precipitated into cold diethyl ether and the solid isolated by filtration. Isolated yield 75–80%.

Characterization data of PEG_n_-*b*-PCAzo_m_, e.g., PEG_113_-*b*-PCAzo_23_: FTIR (KBr disk, cm^−1^): 3141 (Csp^2^–H), 2938 (Csp^3^–H), 1763 (C=O), 1601 (CAr–CAr), 1499, 1474 (N=N), 1248, 1143 (C–O). ^1^H NMR [400 MHz, CDCl_3_, δ, ppm]: 7.89–7.78 (m), 7.64 (s), 7.03–6.89 (m), 5.24 (s), 4.44–4.17 (m), 4.04–3.91 (m), 3.80–3.72 (m), 3.70–3.58 (s, broad), 3.39 (s), 2.17–2.05 (m), 2.01–1.70 (m), 1.59–1.32 (m), 1.20 (s), 1.10–0.97 (m). GPC data: PEG_113_-*b*-PCAzo_23_ *M*_n_ = 12,400, *Ð* = 1.11; PEG_45_-*b*-PCAzo_18_ *M*_n_ = 9530, *Ð* = 1.10.

Characterization data of PEG_n_-*b*-PCAzoOMe_m_, e.g., PEG_113_-*b*-PCAzoOMe_23_: FTIR (KBr disk, cm^−1^): 3138 (Csp^2^–H), 2938(Csp^3^–H), 2105, 1743 (C=O), 1598 (CAr-CAr), 1473 (N=N), 1240, 1148 (C–O). ^1^H NMR [400 MHz, CDCl_3_, δ, ppm]: 7.65 (s), 7.20–1.11 (m), 6.66–6.58 (m), 6.18 (s), 5.23 (s), 4.40–4.31 (m), 4.30–1.18 (m), 4.02–3.93 (m), 3.86–3.76 (m), 3.68–3.57 (s, broad), 3.37 (s), 2.00–1.88 (m), 1.86–1.68 (m), 1.57–1.44 (m), 1.44–1.32 (m), 1.21 (s). GPC data: PEG_113_-*b*-PCAzoOMe_23_ *M*_n_ = 9950, *Ð* = 1.08; PEG_45_-*b*-PCAzoOMe_18_ *M*_n_ = 8030, *Ð* = 1.11.

### 2.2. Preparation of Self-Assemblies in Water

#### 2.2.1. Self-Assembly Procedure

Milli-Q^®^ water was gradually added to a solution of the copolymer (5 mg) in spectroscopic grade THF (1 mL) previously filtered through a 0.2 μm polytetrafluoroethylene (PTFE) filter (Spectrum Laboratories, New Brunswick, NJ, USA). The self-assembly process was followed by measuring the loss of transmitted light intensity at 650 nm due to scattering as a function of water content. When a constant value of turbidity was reached, the resulting suspension was filtered through a 5 μm cellulose acetate filter and dialyzed against water using a Spectra/Por^TM^ dialysis membrane (MWCO, 1 kDa) (Repligen Europe B.V., Breda, The Netherlands) for 2 days to remove THF, changing water 3 times. Water suspensions of the polymeric self-assemblies were diluted with Mili-Q^®^ water to a final concentration 1 mg mL^−1^.

#### 2.2.2. Determination of the Critical Aggregation Concentration and loading of Nile Red

Critical aggregation concentration (CAC) was determined by fluorescence spectroscopy using Nile Red. 87 μL of a solution of Nile Red in dichloromethane (3.7 × 10^−5^ M) was added into a vial and the solvent evaporated. Then, 600 μL of the aqueous suspension of the polymeric aggregates with concentrations ranging from 1.0 × 10^−4^ to 1.0 mg mL^−1^ were added and stirred overnight in orbital shaker. The emission spectrum of Nile Red was registered from 560 to 700 nm while exciting at 550 nm.

#### 2.2.3. Preparation of Rhodamine B loaded Vesicles

Rhodamine B loaded vesicles were prepared as described above by adding a solution of Rhodamine B in water (the concentration was adjusted to have a final feed stock of 5 molecules of Rhodamine B per molecule of block copolymer) to a solution of the copolymer (5 mg) in spectroscopic grade THF (1 mL). Non-encapsulated Rhodamine B was removed during dialysis.

### 2.3. Irradiation Experiments

#### 2.3.1. Irradiation Experiments with UV Light

Samples, either THF solutions at a concentration of 10^−4^ M (referred to the repetitive azobenzene unit) or aqueous suspensions of self-assemblies at a concentration of 1 mg mL^−1^, were irradiated in a quartz UV cuvette, path length of 10 mm for THF solutions and 1 mm for aqueous suspensions, with a compact low-pressure fluorescent lamp Philips PL-S 9 W (Philips Lighting Corporation, Somerset, NJ, USA) emitting between 350 and 400 nm. Irradiance in the sample at 365 nm was 3.5 µW cm^−2^.

#### 2.3.2. Irradiation Experiments with Visible Light

Samples, either THF solutions at a concentration of 10^−4^ M (referred to the repetitive azobenzene unit) or aqueous suspensions of self-assemblies at a concentration of 1 mg mL^−1^, were irradiated in a quartz UV cuvette, path length of 10 mm for THF solutions and 1 mm for aqueous suspensions, with green (530 nm) or red light (625 nm) using a Mightex LCS-0530-15-22 or a Mightex LCS-0625-07-22 high power LED, respectively (Mightex, Toronto, Ontario, Canada). Irradiance in the sample at 530 or 625 nm was 30 µW cm^−2^.

### 2.4. Characterization Techniques and Instrumentation

Fourier transform infrared spectroscopy (FTIR) was applied using a Bruker Tensor 27 FT-IR spectrophotometer (Bruker, Billerica, MA, USA) and KBr disks. NMR experiments were carried out on Bruker Avance spectrometers (Bruker, Billerica, MA, USA) operating at 400 MHz for ^1^H, and 100 MHz for ^13^C, using standard pulse sequences. Chemical shifts are given in ppm relative to TMS and the solvent residual peak was used as internal reference. Relative average molecular masses (*M*_n_) and dispersity (*Đ*) values were determined by size exclusion chromatography (SEC) using a Waters 2695 liquid chromatography system equipped with a Waters 2998 photodiode array and a Waters 2420 evaporation light scattering detectors using two Ultrastyragel columns with pore size of 500 and 10^4^ Å (from Waters) calibrated using poly(methyl methacrylate) standards and THF as solvent (Waters, Milford, MA, USA).

Thermogravimetric analysis (TGA) was performed at 10 °C min^−1^ under nitrogen atmosphere using a SDT 2960 Simultaneous DTA-TGA from TA Instruments (TA Instruments, New Castle, DE, USA). TGA data were given as the onset of the decomposition curve. Differential scanning calorimetry (DSC) was performed using a DSC Q2000 from TA Instruments with samples (approx. 2 mg) sealed in aluminum pans in the range of −50 °C to 120 °C at a scanning rate of 10 °C min^−1^. Temperatures were read at the maximum of the transition peaks, and the glass transition temperature (*T*_g_) was read at the midpoint of the heat capacity increase. The optical textures of the mesophases were studied with an Olympus BH-2 polarizing microscope (Olympus Corporation, Waltham, MA, USA) equipped with a Linkam THMS600 hot stage and a TMS91 cooling system (Linkam Scientific, Tadworth, UK).

UV-vis absorption spectra were recorded in a ATI-Unicam UV4-200 spectrophotometer (ATI_Unicam, Cambridge, UK). THF solutions of 10^−4^ M concentration were recorded on 10 mm quartz cuvettes while 1 mg mL^−1^ water suspensions on 1 mm quartz cuvettes. Fluorescence measurements were performed using a PerkinElmer LS 50 fluorescence spectrophotometer (PE Corporation, Waltham, MA, USA).

High performance liquid chromatography (HPLC) were carried out using a Waters 600 controller pump system with a mixture acetonitrile/100 mM ammonium acetate in water (8:2) as the mobile phase at a 1 mL min^−1^ flow rate, a column Waters Spherisorb 5µm C8 (4.6 × 250 mm, particle size 5 µm and pore size 80 Å) as stationary phase and a Waters 2998 PDA detector at 550 nm (Waters, Milford, MA, USA).

Dynamic light scattering (DLS) measurements were carried out in a Malvern Instrument Nano ZS (Malvern, Worcestershire, UK) using a He-Ne laser with a 633 nm wavelength and a detector angle of 173° at 25 °C. The self-assemblies were measured at 0.10 mg mL^−1^ concentrations and hydrodynamic diameters (*D_h_*) were given as an average of three measurements on each sample to ensure reproducibility.

Transmission Electron Microscopy (TEM) and Transmission Electron Cryo-Microscopy (Cryo-TEM). The morphology of the self-assemblies was studied by TEM using a TECNAI G20 (FEI Company, Waltham, MA, USA) electron microscopes operating at 200 kV. Ten microliters of a 1.0 mg mL^−1^ self-assembly water dispersion was deposited onto a carbon-coated copper grid and the water was removed by capillarity using filter paper. The samples were stained with uranyl acetate removing the excess by capillarity using filter paper. The grids were dried overnight under vacuum. For Cryo-TEM a 3 µL drop of a 1.0 mg mL^−1^ vesicular suspension was placed on a TEM Quantifoil carbon grid, excess of solvent was blotted away with filter paper and the grid freeze-plunged into liquid ethane using a FEI Vitrobot (FEI Company, Waltham, MA, USA). Samples were maintained under liquid nitrogen with a Gatan TEM cryo-holder (FEI Company) and observed in a TECNAI G20 (FEI Company, Waltham, MA, USA) operating at 80 kV.

## 3. Results

### 3.1. Synthesis and Characterization of the Amphiphilic Diblock Copolymers

Azobenzene linear-linear block copolymers were obtained as outlined in Figure 1. Bis-MPA is a 1,3-diol that by reaction with ethyl chloroformate can be easily transformed into a six-membered cyclic carbonate monomer that undergoes ring opening polymerization (ROP) under mild conditions with nucleophilic initiators such as alcohols. The polymerization provides an aliphatic polycarbonate with pendant carboxylate groups from which functionality can be implanted along the backbone. Accordingly, copolymers were obtained by organocatalyzed ROP of 5-methyl-5-propargyloxycarbonyl-1,3-dioxan-2-one (MPC) and further post-polymerization functionalization by copper(I) catalyzed azide-alkyne cycloaddition (CuAAC) with an azobenzene azide. ROP was promoted using poly(ethylene glycol) methyl ether (PEG_n_–OH), acting as a monofunctional macroinitiator, and 1,8-diazabicyclo(5,4,0)undec-7-ene (DBU)/1-(3,5-bis(trifluoromethyl)phenyl)-3-cyclohexylthiourea (TU) as the catalytic system that provides good control over the ROP of different cyclic carbonates and fast polymerization rates [27,28,29]. ROP of MPC was performed in dichloromethane ([MPC]_0_ = 1.0 M) at 40 °C for 8 h using a [MPC]:[TU]:[DBU] = 1:0.05:0.01 ratio. Two poly(ethylene glycol)s (PEG_n_–OH) with average polymerizations degrees (*n*) of 113 and 45 were used while adjusting the [MPC]:[PEG_n_–OH] ratio to get hydrophobic-to-hydrophilic ratios of about 80:20 wt % on the final azobenzene block copolymers. Polymerizations were confirmed by ^1^H NMR by the disappearance of the methylenic protons of the carbonate ring, at δ = 4.71 and 4.22 ppm, and the appearance of a new signal, at 4.25 ppm, corresponding to the polycarbonate backbone (Supplementary Materials, Figures S1 and S2). Average polymerization degrees of the polycarbonate segment, *m*, were determined using ^1^H NMR end group analysis by comparing relative integration of the terminal methoxy group of the PEG block (δ = 3.31 ppm) and the alkynyl side groups of the polycarbonate block (δ = 2.48 ppm). Values of *m* were found to be 23 and 18 when using PEG_113_–OH and PEG_45_–OH. That matched reasonably well with theoretical ones (30 and 23, respectively). Additionally, SEC traces revealed unimodal distributions with *Ð* = 1.06 for PEG_45_-*b*-PC_18_ and 1.08 for PEG_113_-*b*-PC_23_ (Supplementary Materials, Figures S3 and S4).

Functionalization of the polycarbonate block with azides N_3_-Azo or N_3_-AzoOMe, via CuAAC was approached using CuBr/PMDETA in DMF (Figure 1) [22]. Progress of the functionalization was evaluated by ^1^H-NMR from the disappearance of the signal at δ = 2.48 ppm, corresponding this signal to the proton in the propargyl side-group, and by the appearance of a new signal at 7.65 ppm from the proton in the triazole ring (Supplementary Materials, Figures S1 and S2). Complete functionalization of the propargyl groups was confirmed by FTIR, according to the sensibility of the technique, from the disappearance of the Csp–H and Csp‒Csp stretching bands at 3300 cm^−1^ and 2100 cm^−1^ respectively (Supplementary Materials, Figures S5 and S6). SEC mass distribution peaks shifted to lower retention times in comparison to parent polymers PEG_n_-*b*-PC_n_ as expected due to the increase in the molecular mass (Supplementary Materials, Figures S3 and S4), but dispersity values were about the same.

Thermal stability was evaluated by TGA (Supplementary Materials, Figure S7) and data are summarized in Table 1. In general terms, polymers with the longer PEG segment (PEG_113_-*b*-PCAzo_23_ and PEG_113_-*b*-PCAzoOMe_23_) presented decomposition temperatures above 200 °C while those with a shorter one (PEG_45_-*b*-PCAzo_18_ and PEG_45_-*b*-PCAzoOMe_18_) were just below 200 °C. Additionally, the AzoOMe unit decreases the stability in comparison to Azo unit. Evolution of volatiles due to the presence of residual solvents or water was not observed.

Thermal transitions were determined by DSC analysis in the range of −50 °C to 120 °C and relevant data are collected in Table 1 and in the Supporting Information. Neat PEG_45_–OH and PEG_113_–OH are semicrystalline polymers whose melting temperatures were recorded at 49 °C (Δ*H*_m_ = 161 J/g) and 54 °C (Δ*H*_m_ = 158 J/g), respectively (Supplementary Materials, Figures S8 and S9). Only on the first heating scan of PEG_113_-*b*-PCAzo_23_ was an endothermic peak registered at 50 °C (with Δ*H* = 31.8 J g^−1^), which correlates well with melting of PEG. Glass transitions, which have been reported at about −49 °C, were not detected [30]. In all the block copolymers crystallization of the PEG segments was hindered, in particular, for block copolymers of the PEG_45_ series. When inspected by polarizing optical microscopy (POM), PEG_n_-*b*-PCAzo_m_ polymers showed birefringent textures upon heating associated to liquid crystalline properties, as the corresponding homopolymer PCAzo (see structure in Supplementary Materials, Figure S10), which was a liquid crystalline material that exhibited a highly viscous mesophase from 69 °C (melting temperature after cold crystallization, *T*_g_ at 46 °C) to 77 °C (mesophase to isotropic transition temperature) difficult to identify by POM (Supplementary Materials, Figure S10). For the pristine PEG_45_-*b*-PCAzo_18_, some residual PEG crystallization was observed from the first heating curve. However, the melting transition of the PEG block was not observed after cooling and re-heating of the sample suggesting not crystallization. Instead, a glass transition and two endothermic peaks originated from the liquid crystalline polycarbonate block were observed. On the contrary, the heating curve of PEG_113_-*b*-PCAzo_23_ showed the thermal events associated to each block, namely, melting of PEG segment at 45 °C (probably masking the glass transition of PCAzo block), melting of the PCAzo block at 61 °C and the mesophase to isotropic transition at 73 °C. Block copolymers of the PCAzoOMe series were amorphous as they only exhibited a glass transition on the second heating scan attributed to the polycarbonate block.

### 3.2. Self-Assembly in Water and Morphological Analysis

Self-assembly of PEG_n_-*b*-PCAzo_m_ and PEG_n_-*b*-PCAzoOMe_m_ polymers was promoted by the co-solvent method when water was gradually added to THF solutions of the polymers. The process was monitored by turbidimetry recording the decrease of transmitted light through the sample that occurs upon self-assembly (experimental details are given in Supplementary Materials). Morphology and size of the self-assembled structures were determined by transmission electron microscopy (TEM), transmission electron cryomicroscopy (Cryo-TEM), and dynamic light scattering (DLS).

TEM images collected for copolymers of the PEG_113_ series (PEG_113_-*b*-PCAzo_23_ and PEG_113_-*b*-PCAzoOMe_23_), which comprise about 75% hydrophobic content in mass percentage, showed the formation of spherical micelles (Supplementary Materials, Figure S11) with the azobenzene segments forming the compact core. Average hydrodynamic diameters, *D_h_*, measured by DLS were 24 nm for PEG_113_-*b*-PCAzo_23_ and 30 nm for PEG_113_-*b*-PCAzoOMe_23_, matching with the values estimated from the TEM images (Supplementary Materials, Figure S11). Critical aggregation concentrations (CAC) were determined by fluorescence spectroscopy using Nile Red as a probe (experimental details are given in Supplementary Materials), obtaining values of 17 and 22 μg mL^−1^ for PEG_113_-*b*-PCAzo_23_ and PEG_113_-*b*-PCAzoOMe_23_ (Supplementary Materials, Figure S12).

For block copolymers of the PEG_45_ series (PEG_45_-*b*-PCAzo_18_ and PEG_45_-*b*-PCAzoOMe_18_) having approx. 85% hydrophobic mass percentage, vesicles were visualized by TEM and Cryo-TEM (Figure S13) with the azobenzene hydrophobic segments confined inside the vesicle’s membrane hydrophobic region. A *D_h_* of 350 nm for PEG_45_-*b*-PCAzo_18_ and 220 nm for PEG_45_-*b*-PCAzoOMe_18_ were determined by DLS (Supplementary Materials, Figure S13). The CACs for PEG_45_-*b*-PCAzo_18_ and PEG_45_-*b*-PCAzoOMe_18_ were found to be 32 and 33 μg mL^−1^, respectively (Supplementary Materials, Figure S14). The increase of CAC on increasing the hydrophobicity of the BCs, also associated to a change from micelles to vesicles, has been observed for other BCs [31] and could be related with the shorter length of the PEG block on the vesicles. The longer PEG chain length on the micellar self-assemblies will lead to a higher coverage of the micelle core increasing their stability and, consequently, displaying lower CAC values [32].

### 3.3. Light Responsiveness of PEG_n_-b-PCAzo_m_ Self-Assemblies

UV-Vis spectra of the PEG_n_-*b*-PCAzo_m_ series polymers was registered both in the THF solution, at a concentration of 10^−4^ M relative to the azobenzene moieties, and the self-assemblies aqueous dispersions, at a concentration of 1 mg of BC per mL. The solution spectra displayed two absorption bands arising from the *trans*-azobenzene isomer, a strong one located at 360 nm corresponding to the π-π* transition and a weak one centered at 450 nm which corresponds to the forbidden n-π* transition (Supplementary Materials, Figure S15). After 15 s exposure to a UV light lamp (Irradiance in the sample of 3.5 µW cm^−2^ at 365 nm), a drastic decrease of the absorbance at 360 nm accompanied by the apparition of a new less intense π–π* band centered at 330 nm corresponding to *cis-*azobenzene unit was observed, alongside with an increase in the absorbance at 450 nm due to the *trans*-*cis* photoisomerization. These spectral features agree well with those reported for linear-dendritic BCs decorated with the same azobenzene moiety [19].

**Figure 2:**
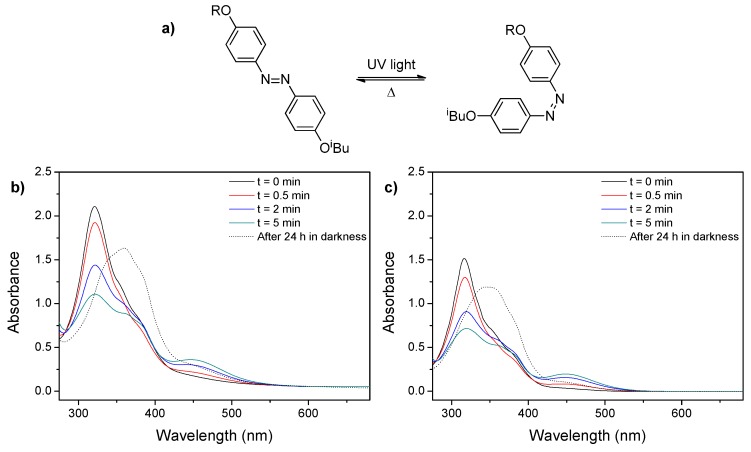
Schematic representation of the isomerization of azobenzene moieties located in the side-chain of the hydrophobic block. (**a**) UV-Vis spectra of 1 mg mL^−1^ self-assemblies water suspensions upon UV light illumination for different times and subsequent storage at room temperature for 24 h in the dark: (**b**) PEG_45_-*b*-PCAzo_18_ vesicles and (**c**) PEG_113_-*b*-PCAzo_23_ micelles.

The spectra of the self-assemblies (both micelles and vesicles) aqueous dispersions showed a blue shift of the π–π* band maximum from 360 nm to 320 nm indicative of the prevalent formation of H-aggregates of azobenzene units (Figure 2). Moreover, two weak shoulders were observed, one at 360 nm associated to the non-aggregated azobenzenes and one at 375 nm arising the formation of J-aggregates [19,33,34,35,36,37]. Upon UV illumination, a decrease on the intensity of the π–π* band with a concurrent increase in the absorbance at 450 nm was monitored and associated to the *trans*-*cis* photoisomerization and changes of the azobenzene aggregation. No further evolution on the spectra was observed after 5 min of illumination inferring that a photostationary state was reached at this point. After storing the irradiated sample at room temperature for 24 h in the dark, the initial spectra were not recovered as the broad π–π* band was centered at 360 nm (instead of 320 nm), suggesting an azobenzene aggregation different to the observed on the non-irradiated self-assemblies.

**Figure 3:**
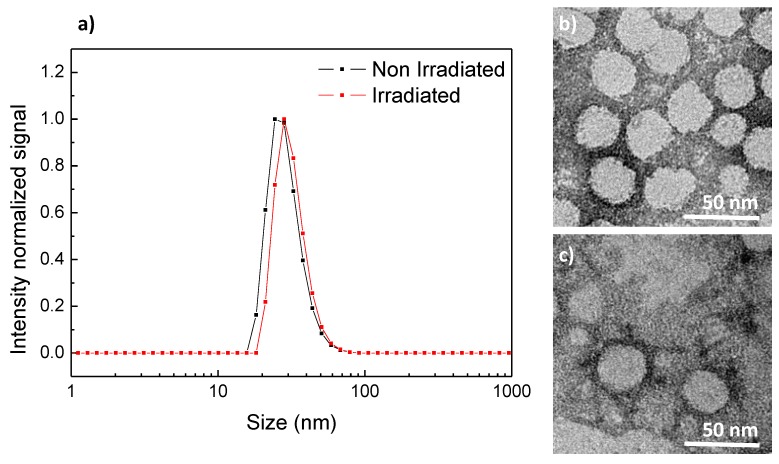
(**a**) Dynamic light scattering (DLS) traces and TEM images (**b**) before and (**c**) after 10 min UV irradiation of PEG_113_-*b*-PCAzo_23_ self-assemblies.

Once the *trans*-*cis* photoisomerization of the 4-isobutyloxyazobenzene moieties confined into the hydrophobic regions of the self-assemblies was corroborated, morphological changes of the micelles and vesicles after UV light illumination were studied by TEM and DLS. After 10 min of exposure to UV light PEG_113_-*b*-PCAzo_23_ micelles were still visible across the TEM grid, although its morphology was less defined and was accompanied by organic material without a defined morphology, in a similar way to previous reported azobenzene light-responsive micelles [33] (Figure 3b,c). A slight increase in the size of the micelles was measured by DLS, with *D_h_* going up from 24 nm to 30 nm (Figure 3a).

**Figure 4:**
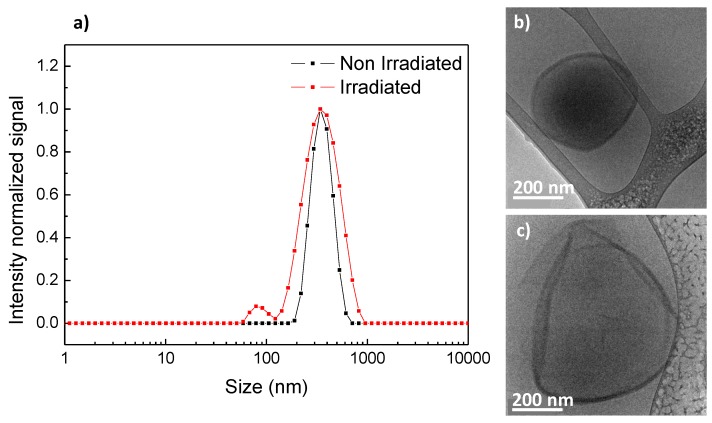
(**a**) DLS traces and Cryo-TEM images (**b**) before and (**c**) after 10 min UV light irradiation of PEG_45_-*b*-PCAzo_18_ self-assemblies.

Upon UV illumination, vesicles from PEG_45_-*b*-PCAzo_18_ appeared distorted and wrinkled in the TEM images as in the related linear-dendritic block copolymers previously described with this 4-isobutyloxyazobenzene unit [19,20]. These vesicles were found to coexist with smaller vesicles that were not observed in non-irradiated samples (Supplementary Materials, Figure S16). Alteration of the vesicles was confirmed by Cryo-TEM inspection, where the continuous and smooth vesicle became non-continuous and folded (Figure 4b,c and Figure S17). In accordance, by DLS, an increase in the size dispersity of the self-assemblies and the appearance of a second smaller size population was observed (Figure 4a).

### 3.4. Light Responsiveness of PEG_n_-b-PCAzoOMe_m_ Self-Assemblies

The UV-vis spectra of tetra-*ortho*-substituted azobenzene polymers, PEG_45_-*b*-PCAzoOMe_18_, and PEG_113_-*b*-PCAzoOMe_23_ in solution showed (Supplementary Materials, Figure S18) a main band corresponding to the π–π* electronic transition was centered at 320 nm and a second absorption band due to n-π* transition was located at 470 nm. Due to the non-planar molecular geometry of the tetra-*ortho* substituted *trans*-azobenzene moiety, this n-π* transition absorption band is significantly more intense and red-shifted compared to PCAzo series (470 nm versus 450 nm) [38]. This n-π* band is extended beyond 600 nm and, consequently, the photoisomerization could be induced with red light [12].

The *trans*-*cis* isomerization of the tetra-*ortho*-substituted azobenzene units was induced using a LED of 625 nm or 530 nm wavelength (Irradiance in the sample of 30 µW cm^−2^). When BCs THF solutions were exposed to 625 nm light (see experimental details in experimental section) a photostationary state was reached after 40 min. Spectra showed a remarkable decrease in the intensity of π–π* band and a blue shift of the n-π* band, from 470 to 445 nm, attributed to the *trans*-to-*cis* photoisomerization of the photoactive units (Supplementary Materials, Figure S18). The same trend was observed under 530 nm irradiation although in a shorter timescale, i.e., the photostationary state was reached only after 30 s, due to a better matching of the absorption band and the light wavelength. Additionally, the decrease in the intensity of the π–π* band was more acute and the n-π* band was shifted up to 435 nm. The evolution of spectra upon illumination showed the presence of two isosbestic points at 390 and 460 nm giving evidence of an equilibrium between two different species during the photoisomerization without the occurrence of other competitive processes [39]. The *cis*-azobenzene fraction (γ), on the photostationary state can be estimated from the absorbance at 330 nm using the expression γ = 1.05∙(1 − A/A_0_) being A_0_ and A absorbance before and after irradiation [18,40]. Under 625 nm light the *cis* content was approx. 30% at the photostationary state for both copolymers, while under 530 nm light the *cis* content raised up to approx. 55%.

**Figure 5:**
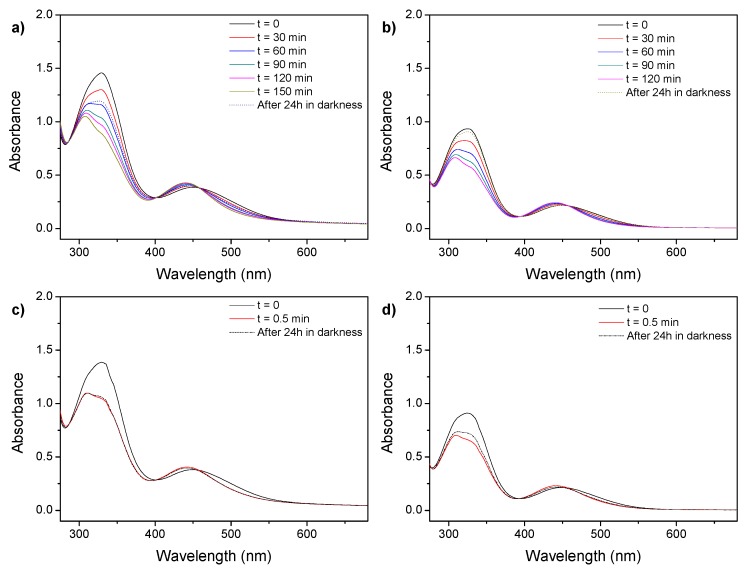
UV-Vis spectra of a 1 mg mL^−1^ self-assemblies water suspensions of (**a**) PEG_113_-*b*-PCAzoOMe_23_ and (**b**) PEG_45_-*b*-PCAzoOMe_18_ under 625 nm light illumination for different times and subsequent storage for 24 h in the dark and (**c**) PEG_113_-*b*-PCAzoOMe_23_ and (**d**) PEG_45_-*b*-PCAzoOMe_18_ under 530 nm light illumination for different times and subsequent storage for 24 h in the dark.

Compared to solution, UV-Vis spectra of the self-assembled structures showed a shift of the n-π* band to higher frequencies, from 470 to 450 nm (Figure 5). Under visible light illumination both micelles and vesicles showed similar behavior. When self-assemblies were illuminated with 625 nm light, the photostationary state was reached in irradiation times about 120–150 min, which are longer illumination times when compared to solution, probably due to the blue-shifting of the n-π* band with respect to THF solutions and the confinement of azobenzene moieties in the hydrophobic regions of the self-assemblies (Figure 5a,b). At the photostationary state the *cis* content was similar for both micelles and vesicles, about 40%. Once the light was turn off and the irradiated samples were stored in the dark for 24 h, the initial situation was only partially recovered, as a *cis* content of approx. 20% and 5% was found for micelles and vesicles, respectively (Figure 5a,b).

By irradiating the aqueous dispersions with 530 nm light, the photostationary states were reached at the same timescale than in solution (Figure 5c,d) but rendering lower *cis* contents in comparison to those obtained with 625 nm light: 26% and 29% for micelles and vesicles, respectively. It was remarkable the low thermal reversibility of the light induced changes after storing the samples 24 h in the dark for both types of self-assemblies (Figure 5c,d).

**Figure 6:**
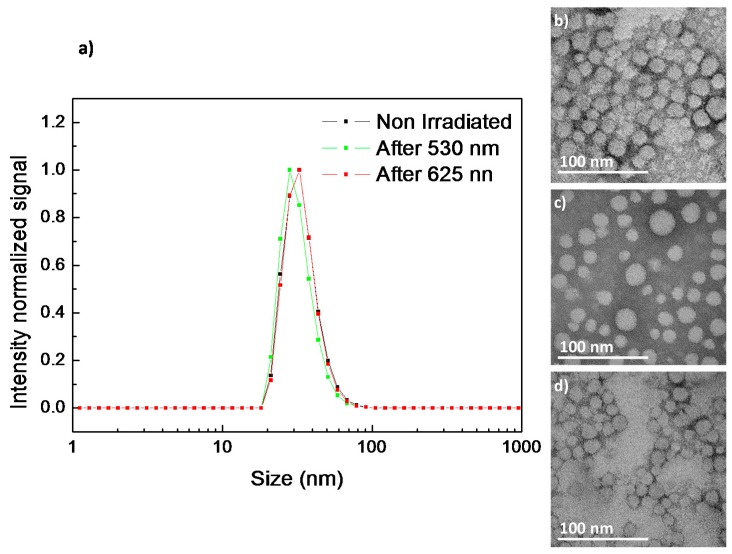
DLS traces (**a**) and TEM images before (**b**) and after 2 h 625 nm irradiation (**c**) and after 5 min 530 nm light irradiation (**d**) of PEG_113_-*b*-PCAzoOMe_23_ micelles.

According to TEM images and DLS analysis, micelles of PEG_113_-*b*-PCAzoOMe_23_ remained relatively stable after 2 h under 625 nm light exposure (Figure 6a,c). However, they were affected by the action of 530 nm light. After 5 min, a slight modification in size was registered by DLS, with the average dimeter decreasing from 30 to 27 nm (Figure 6a). On the TEM images, the unmodified micelles coexisted with smaller ones (Figure 6d).

**Figure 7:**
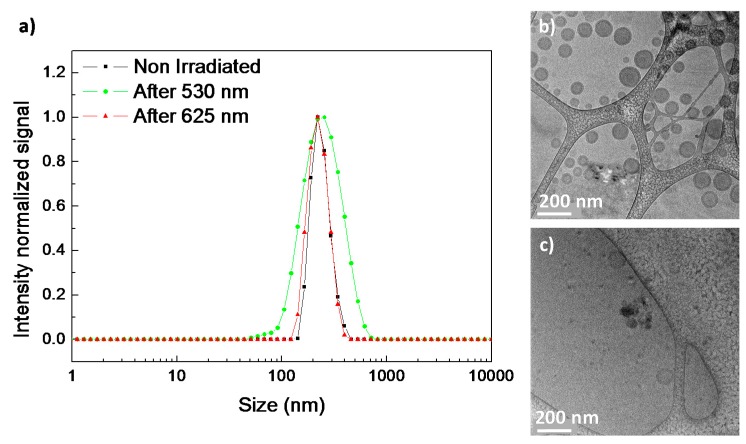
DLS traces before and after 120 min under 625 nm light and after 5 min under 530 nm light (**a**) Cryo-TEM images before (**b**) and after 5 min 530 nm light irradiation (**c**) of PEG_45_-*b*-PCAzoOMe_18_ vesicles.

When the vesicles dispersion of PEG_45_-*b*-PCAzoOMe_18_ was exposed to 625 nm light for up to 2 h, the effect of illumination on the self-assemblies was negligible according to TEM images (Supplementary Materials, Figure S19) and DLS measurements (Figure 7a). However, after 5 min under 530 nm light a remarkable increase in the size dispersity of the self-assemblies was monitored by DLS (Figure 7a) as a consequence of the *trans*-*cis* isomerization. Disruption of the vesicle morphology was confirmed by TEM (Supplementary Materials, Figure S19) and Cryo-TEM (Figure 7c), as the number of vesicle diminishes after 530 nm light irradiation.

### 3.5. Encapsulation and Light-Induced Release of Molecular Probes

**Figure 8:**
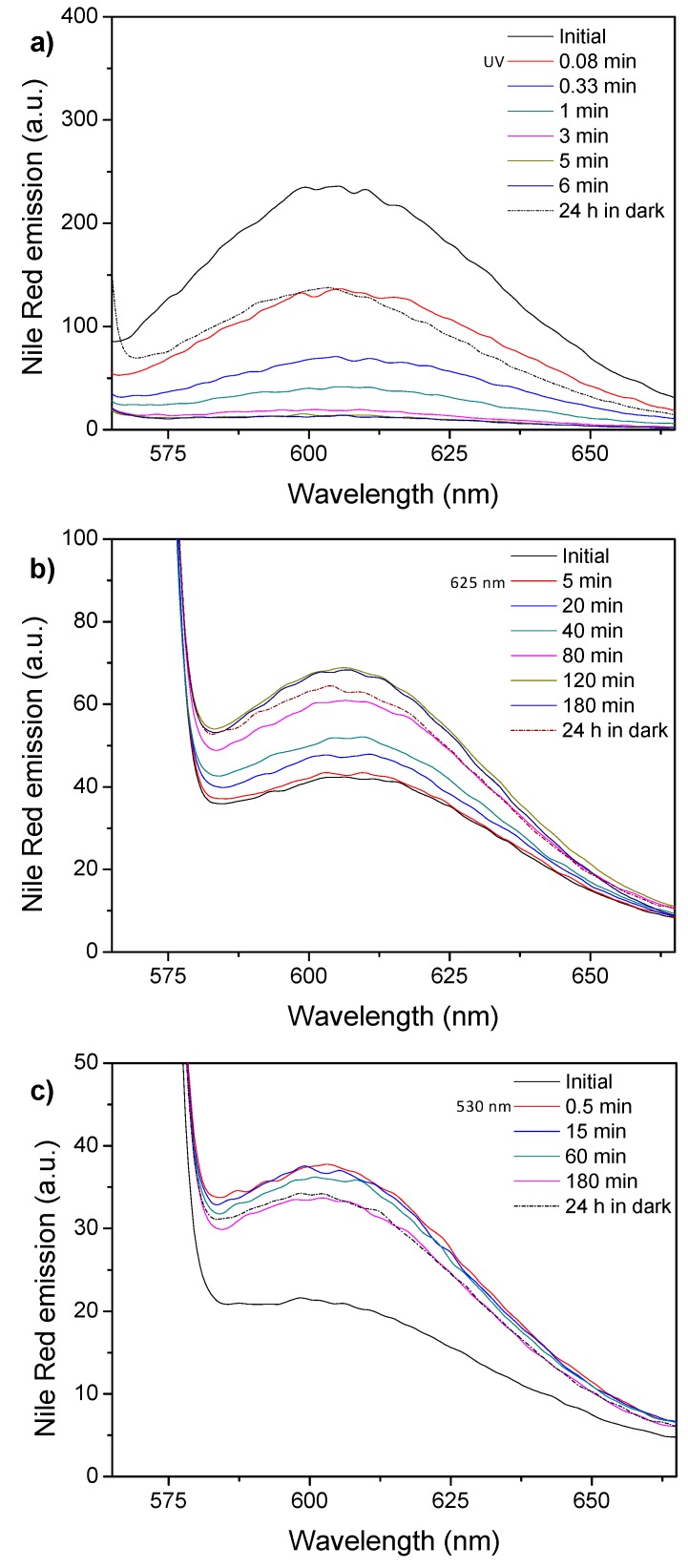
Emission spectra (excitation wavelength 550 nm) of the Nile Red loaded micelles recorded after light illumination and for different time intervals and subsequent storage for 24 h in the dark for (**a**) PEG_113_-*b*-PCAzo_23_ micelles under UV light (Irradiance of 3.5 µW cm^−2^ at 365 nm), (**b**) PEG_113_-*b*-PCAzoOMe_23_ micelles under 625 nm light (Irradiance of 30 µW cm^−2^ at 625 nm), and (**c**) PEG_113_-*b*-PCAzoOMe_23_ micelles under 530 nm light (Irradiance of 30 µW cm^−2^ at 530 nm).

After having evaluated the light-response of micelles and vesicles, their potential capability as light responsive, in particular visible responsive, nanocarriers were tested by using fluorescent molecular probes. Micelles were loaded at the internal core with the well-known hydrophobic fluorescent probe, Nile Red and the changes on the emission intensity registered at different light exposure times (Figure 8). Nile Red loaded PEG_113_-*b*-PCAzo_23_ micelles showed an intense and broad emission band located at 610 nm (when exciting at 550 nm) that sharply decreased during the UV illumination process (Figure 8a). The emission was only partially recovered after 24 h in dark, which seems to evidence that Nile Red is released from the hydrophobic micelle core [20].

Nile Red and AzoOMe n-π*absorption bands are partially overlapped, being this overlapping smaller for the *cis* isomer as its n-π*absorption band is blue-shifted in comparison to the *trans* one (Figure 5). Moreover, the *trans*-isomer can also absorb the light emitted by Nile Red. Under exposure to 625 nm light, the emission of Nile Red loaded PEG_113_-*b*-PCAzoOMe_23_ micelles increased steadily in the same time interval that *trans*-*cis* isomerization takes place, because Nile Red emission was enhanced due to the decrease of the percentage of the *trans* isomer. After 120 min under 625 nm light irradiation, once the photostationary state was reached, Nile Red emission remained constant, which seems to evidence that it was not released to the surrounding media by the effect of 625 nm irradiation (Figure 8b). After 24 h in the dark, the thermal *cis*-to-*trans* relaxation takes place and the percentage of *trans* isomer increases. Consequently, the emission of Nile Red decreases (Figure 8b). Under exposure to 530 nm light for 30 s an initial increase in Nile Red emission was observed due to the *trans*-to-*cis* isomerization. However, under longer exposure times (photostationary state was detected at around 30 s, see above), a steady decrease in Nile Red emission was recorded that might be a consequence of Nile Red release to the aqueous media (Figure 8c). Light of 530 nm wavelength seems to induce not only morphological changes in the micelles but also the release of hydrophobic molecular cargoes encapsulated in the micelles core. After 24 h in the dark, Nile Red emission remained almost constant, in accordance with the low thermal reversibility observed under these experimental conditions (see above). Cargo release profiles of the Nile Red loaded micelles are available at Supplementary Materials, Figure S20.

**Figure 9:**
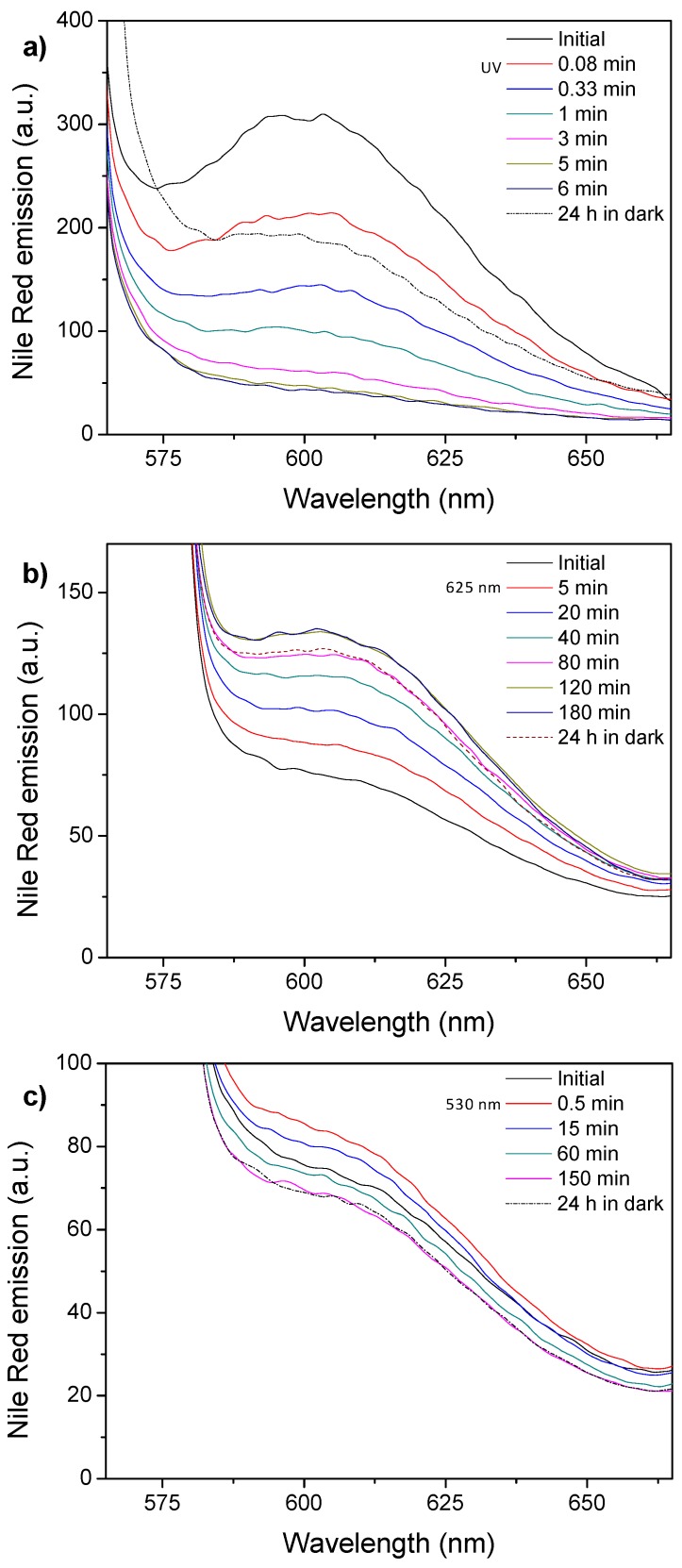
Emission spectra (excitation wavelength 550 nm) of the Nile Red loaded vesicles recorded after light illumination and for different time intervals and subsequent storage for 24 h in the dark for (**a**) PEG_45_-*b*-PCAzo_18_ vesicles under UV light (Irradiance of 3.5 µW cm^−2^ at 365 nm), (**b**) PEG_45_-*b*-PCAzoOMe_18_ vesicles under 625 nm light (Irradiance of 30 µW cm^−2^ at 625 nm), and (**c**) PEG_45_-*b*-PCAzoOMe_18_ vesicles under 530 nm light (Irradiance of 30 µW cm^−2^ at 530 nm).

Encapsulation and light induced release was also tested for vesicles using either hydrophilic or hydrophobic fluorescent probes, Nile Red and Rhodamine B, respectively. Nile Red loaded PEG_45_-*b*-PCAzo_18_ vesicles showed similar features under UV light exposure to those described for the corresponding micelles, which is compatible with partial release of Nile Red from the membrane to the aqueous surrounding media under UV light irradiation (Figure 9a). Emission of Nile Red loaded PEG_45_-*b*-PCAzoOMe_18_ vesicles increased under 625 nm light in the same time interval where *trans*-*cis* takes place (Figure 9b). Again, when the photostationary state was reached after 120 min, a constant emission value was measured. As in the case of micelles, irradiation with 625 nm seems to be not efficient to provoke the release of the encapsulated hydrophobic cargo. Under 530 nm illumination it is observed again the same behavior as in PEG_113_-*b*-PCAzoOMe_23_ micelles, an initial increase for the first 30s during *trans-cis* isomerization and then a continuous decrease, associated to Nile Red release to aqueous media (Figure 9c). Cargo release profiles of the Nile Red loaded vesicles are available in Supplementary Materials, Figure S21.

**Figure 10:**
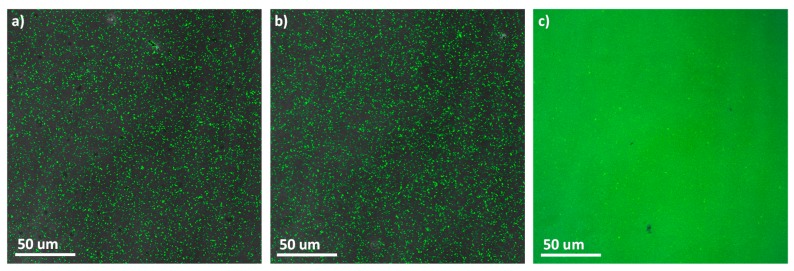
Fluorescence microscopy images of Rhodamine B loaded PEG_45_-*b*-PCAzoOMe_18_ vesicles before (**a**) and after 2 h 625 nm light irradiation (**b**) and after 5 min 530 nm light irradiation (**c**).

Vesicles of PEG_45_-*b*-PCAzo_18_ and PEG_45_-*b*-PCAzoOMe_18_ were loaded with Rhodamine B which, given its hydrophilic character, would be trapped inside the aqueous cavity of the vesicle. The encapsulation and light induced release of Rhodamine B was monitored by confocal microscopy. Images of the initial samples showed fluorescence dots on a dark background verifying the entrapment of the fluorescent probe inside the vesicles (Supplementary Materials, Figure S22 and Figure 10a). The number of encapsulated molecules was determined by HPLC, being 0.1 molecules of Rhodamine B per block copolymer chain (experimental details are given in Experimental Section). Images collected for vesicles of PEG_45_-*b*-PCAzo_18_ after being exposed for 10 min to UV light exhibited a fluorescent background as a consequence of the photoinduced release of Rhodamine B from the vesicles inner cavity to the surrounding media (Supplementary Materials, Figure S22) [19,41]. For vesicles of PEG_45_-*b*-PCAzoOMe_18_, differences were very clear depending on the illumination wavelength. After 2 h at 625 nm light illumination, the fluorescence dots were still visible and the background remained dark, that is to say, the isomerization induced by 625 nm illumination did not stimulate the release of the encapsulated Rhodamine B (Figure 10b). However, in the sample illuminated with 530 nm light for 5 min, the fluorescence is extended all over the background, being still visible some fluorescence dots (Figure 10c).

## 4. Conclusions

A series of amphiphilic diblock copolymers with the light responsive units 4-isobutyloxyazobenzenes (Azo, UV light responsive) or 2,2′,5,5′-tetramethoxyazobenzene (AzoOMe, visible light responsive) have been synthesized by combination of ROP and CuAAC. The length of the hydrophilic segment has influence in the morphology of the self-assemblies, micelles are formed for PEG_113_-*b*-PCAzo_23_ and PEG_113_-*b*-PCAzoOMe_23_, while PEG_45_-*b*-PCAzo_18_ and PEG_45_-*b*-PCAzoOMe_18_ self-assemble into vesicles. Micelles have been loaded with a hydrophobic fluorescent probe, Nile Red, and vesicles with Nile Red and a hydrophilic fluorescent probe, Rhodamine B.

Changes in the morphology of the assemblies from PEG_45_-*b*-PCAzo_18_ and PEG_113_-*b*-PCAzo_23_ can be induced with UV light irradiation, triggering the release of encapsulated Nile Red and Rhodamine B. After 625 nm light irradiation, PEG_113_-*b*-PCAzoOMe_23_ and PEG_45_-*b*-PCAzoOMe_18_ self-assemblies suffer no morphological changes and consequently the release of encapsulated fluorescent probes is not possible. Nonetheless, irradiation with 530 nm light induces morphological changes, and those changes trigger the release of Nile Red and Rhodamine B. Thus, we conclude that 2,2′,5,5′-tetramethoxyazobenzene may be a promising candidate to substitute classical UV sensitive azobenzenes for light-responsive nanocarriers.

## Figures and Tables

**Table 1:** Thermal stability and transition temperatures of Azobenzene block copolymers.

Polymer	Hydrophobic/hydrophylic (wt %) ^1^	TGA (°C) ^2^	*T*_g_ (°C) ^3^	*T*_m_ (°C) [Δ*H*_m_ (J g^−1^)] ^4^	*T*_M-I_ (°C) [Δ*H*_M-I_ (J g^−1^)] ^5^
PEG_45_-*b*-PCAzo_18_	84/16	195	31	63 [12.9]	73 [4.5]
PEG_113_-*b*-PCAzo_23_	73/27	258	-	45 [17.5], 61 [-] ^6^	73 [10.6] ^6^
PEG_45_-*b*-PCAzoOMe_18_	85/15	185	39	-	-
PEG_113_-*b*-PCAzoOMe_23_	75/25	226	19	-	-

^1^ Hydrophobic/hydrophilic weight ratio in % ^2^ Decomposition temperature determined by thermogravimetric analysis (TGA) given in °C at the onset of the weight loss curve. ^3^ Glass transition temperature (*T*_g_) of the azobenzene polycarbonate determined by DSC during the second heating scan at 10 °C min^−1^. ^4^ Melting temperature (*T*_m_) and associated melting enthalpy (Δ*H*_m_) determined by differential scanning calorimetry (DSC) during the second heating scan at 10 °C min^−1^. ^5^ Mesophase-to-isotropic liquid transition temperature (*T*_M-I_) and associated enthalpy (Δ*H*_M-I_) calculated from the second heating scan at 10 °C min^−1^. ^6^ Peaks at 61 and 73 °C overlap, the combined enthalpy value was 10.6 J g^−1^.

## Supplementary Materials

The following are available online at https://www.mdpi.com/2073-4360/11/12/2060/s1, Scheme S1. General Synthesis of Azides N3-Azo y N3-AzoOMe. Figure S1. 1H-NMR (400 MHz, CDCl3) spectra of (from top to bottom) PEG45-b-PC18, PEG45-b-PCAzo18, and PEG45-b-PCAzoOMe18. Figure S2. 1H-NMR (400 MHz, CDCl3) spectra of (from top to bottom) PEG113-b-PC23, PEG113-b-PCAzo23, and PEG113-b-PCAzoOMe23. Figure S3. SEC traces for PEG45-OH, PEG45-b-PC18, PEG45-b-PCAzo18, and PEG45-b-PCAzoOMe18. Figure S4. SEC traces for PEG113-OH, PEG113-b-PC23, PEG113-b-PCAzo23, and PEG113-b-PCAzoOMe23. Figure S5. PEG45-b-PC18, PEG45-b-PCAzo18, and PEG45-b-PCAzoOMe18 FTIR spectrum (KBr disk) (a), and zoom to Csp-H zone (b) and C≡C zone (c). Figure S6: PEG113-b-PC23, PEG113-b-PCAzo23, and PEG113-b-PCAzoOMe23 FTIR spectrum (KBr disk) (a), and zoom to Csp-H zone (b) and C≡C zone (c). Figure S7. TGA curves registered at 10 °C min^−1^ heating rate under nitrogen atmosphere. Figure S8. DSC curves registered on cooling (above) and subsequent heating (below) at a 10 °C min^−1^ scanning rate of PEG45-OH, PEG45-b-PCAzo18 and PEG45-b-PCAzoOMe18. Figure S9. DSC curves registered on cooling (above) and subsequent heating (below) at a 10 °C min^−1^ scanning rate of PEG113-OH, PEG113-b-PCAzo23, and PEG113-b-PCAzoOMe23. Figure S10. Structure of PCAzo (above), DSC curves registered at a 10 °C min^−1^ scanning rate (middle) and POM image (below) captured at 73 °C for homopolymer PCAzo. From the DSC scans, it was deduced that the isotropic liquid the mesophase vitrifies on cooling. On subsequent heating, the glass transition was measured at 46 °C followed by a cold crystallization process. Two endothermic transitions at 69 °C (ΔH = 5.4 kJ per mole of repeating unit) and 77 °C (ΔH = 2.5 kJ per mole of repeating unit) were registered corresponding to melting of the crystalline fraction and to the mesophase-to-isotropic liquid transition, respectively. No clearly identifiable textures were observed even after prolonged thermal annealing of the sample. Figure S11. Analysis of the self-assembled structures of PEG113 amphiphilic block copolymers series. (a) DLS distribution curves of PEG113-b-PCAzo23 and PEG113-b-PCAzoOMe23. TEM image of (b) PEG113-b-PCAzo23 and(c) PEG113-b-PCAzoOMe23. Figure S12. Normalized fluorescence emission of Nile Red at 606 nm (λexc = 550 nm) versus the PEG113-b-PCAzo23 and PEG113-b-PCAzoOMe23 concentration. CAC was determined from the intersection of the two extrapolated lines. Figure S13. Analysis of the self-assembled structures of PEG45 amphiphilic block copolymers series. (a) DLS distribution curves of PEG45-b-PCAzo18 and PEG45-b-PCAzoOMe18. TEM images of (b) PEG45-b-PCAzo18 and (c) PEG45-b-PCAzoOMe18. Cryo-TEM image of (d) PEG45-b-PCAzo18 and (e) PEG45-b-PCAzoOMe18. Figure S14. Normalized fluorescence emission of Nile Red at 606 nm (λexc = 550 nm) versus the PEG45-b-PCAzo18 and PEG45-b-PCAzoOMe18 concentration. CAC was determined from the intersection of the two extrapolated lines. Figure S15. UV-Vis spectra of a 10-4 M (referred to the repetitive azobenzene unit) solutions PEG113-b-PCAzo23 and PEG45-b-PCAzo18 in THF, before and after 15 s UV illumination. Figure S16. TEM images of PEG45-b-PCAzo18 self-assemblies before (a) and after 10 min low intensity UV light irradiation (b). Figure S17. Cryo-TEM images of PEG45-b-PCAzo18 self-assemblies before (a and b) and after 10 min low intensity UV light irradiation (c and d). Figure S18. UV-Vis spectra of a 10-4 M (referred to the repetitive azobenzene unit) PEG113-b-PCAzoOMe23 and PEG45-b-PCAzoOMe18 solution in THF and photostationary state reached after 40 min under 625 nm light and after 30 s under 530 nm light. Figure S19: TEM images of PEG45-b-PCAzoOMe18 self-assemblies before (a), after 2 hours 625 nm light irradiation (b), and after 5 min 530 nm light irradiation (c). Figure S20. Cargo release profiles of the Nile Red loaded micelles for (a) PEG113-b-PCAzo23 micelles under UV light, (b) PEG113-b-PCAzoOMe23 micelles under 625 nm light, and (c) PEG113-b-PCAzoOMe23 micelles under 530 nm. Figure S21. Cargo release profiles of the Nile Red loaded micelles for (a) PEG45-b-PCAzo18 vesicles under UV light, (b) PEG45-b-PCAzoOMe18 vesicles under 625 nm light, and (c) PEG45-b-PCAzoOMe18 vesicles under 530 nm. Figure S22. Fluorescence microscopy images of Rhodamine B loaded PEG45-b-PCAzo18 vesicles before (a) and after 10 min low intensity UV light irradiation (b).
