# Supplementary material for: Polymeric Self-Assemblies Based on tetra-ortho-Substituted Azobenzene as Visible Light Responsive Nanocarriers
Source: Polymers (Basel). 2019 Dec 11;11(12):2060. doi: 10.3390/polym11122060 (PMC6960964; doi:10.3390/polym11122060)
Supplement: Supplementary file 1 [file polymers-11-02060-s001.pdf]

Supplementary Materials

Polymeric Self-Assemblies Based on tetra-*ortho*-Substituted Azobenzene as Visible Light Responsive Nanocarriers

Alejandro Roche [1], Luis Oriol [1], Rosa M. Tejedor [1,2,*] and Milagros Piñol [1,*]

1. Synthesis and characterization of N_3-Azo and N_3-AzoOMe

Azide azobenzenes were synthesized as illustrated in Scheme S1 from the corresponding 4-hydroxyazobencenes *via* Mitsunobu reaction with 6-azidohexan-1-ol. -4-Isobutyloxy-4′-hydroxyazobenzene [1], 2,2′,5,5′-tetramethoxy-4-hydroxy-azobenzene [2] and 6-azidohexan-1-ol [3] were synthesized according to previously reported procedures.

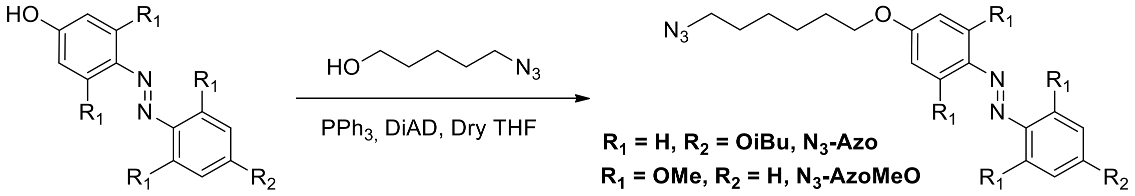

Scheme S1. General Synthesis of Azides N_3-Azo y N_3-AzoOMe

1.1. Synthesis and characterization of 4-isobutyloxy-4′-(6-azidohexyl-1-oxy)azobenzene (N_3-Azo)

4-Isobutyloxy-4′-hydroxyazobenzene (1.50 g, 5.54 mmol), 6-azidohexanol (786 mg, 5.54 mmol) and diisopropyl azodicarboxylate (DIAD) (1.12 g, 5.54 mmol) were dissolved in dry THF (50 mL) under Ar atmosphere and cooled down in an acetone-ice bath. Then, a triphenylphospine (PPh_3) (1.45 g, 5.54 mmol) solution in anhydrous THF (10 mL) was added dropwise. The reaction mixture was stirred for 24 h. Solvent was evaporated to dryness and the product purified by recrystalization in ethanol. Product was isolated by filtration as a pale orange solid. Yield 80%. FTIR (KBr disk, cm^{-1}): 3050 (Csp^2-H), 2938 (Csp^3-H), 2102 (N_3), 1560 (CAr-CAr), 1471 (N=N), 1239 (C-O). ^1H NMR [400 MHz, $CDCl_3$, δ, ppm]: 7.94 - 7.79 (m, 4H), 7.07 - 6.91 (m, 4H), 4.03 (t, *J* = 6.4 Hz, 2H), 3.80 (d, *J* = 6.6 Hz, 2H), 3.29 (t, *J* = 6.8 Hz, 2H), 2.17 - 2.06 (m, 1H), 1.90 - 1.78 (m, 2H), 1.72 - 1.58 (m, 2H), 1.57 - 1.38 (m, 4H), 1.05 (d, *J* = 6.8 Hz, 6H). ^{13}C NMR [100 MHz, $CDCl_3$, ppm]: 161.45, 161.15, 147.15, 147.05 124.32, 114.82. 114.18, 74.81, 68.16, 51.51, 29.22, 28.93, 28.42, 26.65, 25.80, 19.37.

1.2. Synthesis and characterization of 2,2′,5,5′-tetramethoxy-4-oxo-(6′′-azidohexyl-1-oxy) azobenzene (N_3-AzoOMe)

2,2′,5,5′-Tetramethoxy-4-hydroxyazobenzene, (400 mg, 1.26 mmol), DPTS (146 mg, 0.51 mmol) and 6-azidohexanoic acid (239 mg, 1.52 mmol) were dissolved in dry dichloromethane (10

mL) under Ar atmosphere. The flask was cooled in an acetone-ice bath. Then, EDC (290 mg, 1.52 mmol) was added under Ar atmosphere. After half an hour, the ice bath was removed and the reaction was stirred for 72 h. Crude reaction was diluted with dichloromethane (100 mL) and washed with water (2 × 100 mL) and brine (100 mL). The organic phase was dried over anhydrous $MgSO_4$, filtered off and evaporated. The residue was purified by silica column chromatography, using dichloromethane/ethyl acetate (8/2) as eluent. Yield 45%. FTIR (KBr, υmax/cm^{-1}): 3009 (Csp^2-H), 2942 (Csp^3-H), 2092 (N_3), 1581 (CAr-CAr), 1472 (N=N), 1255 (C-O). NMR ^1H [400 MHz, $CDCl_3$, δ (ppm)]: 7.18 (t, J = Hz, 1H), 6.65 (d, J = Hz, 2H), 6.21 (s, 2H), 4.01 (t, J = Hz, 2H), 3.87 (s, 6H), 3.84 (s, 6H), 3.30 (t, J = Hz, 2H), 1.90 - 1.75 (m, 2H), 1.73 - 1.60 (m, 2H), 1.60 - 1.39 (m, 4H). NMR ^{13}C [100 MHz, $CDCl_3$, δ (ppm)]: 161.03, 154.49, 152.28, 134.82, 128.71, 128.52, 105.2, 91.92, 67.75, 56.62, 51.33, 29.06, 28.75, 26.49, 25.64.

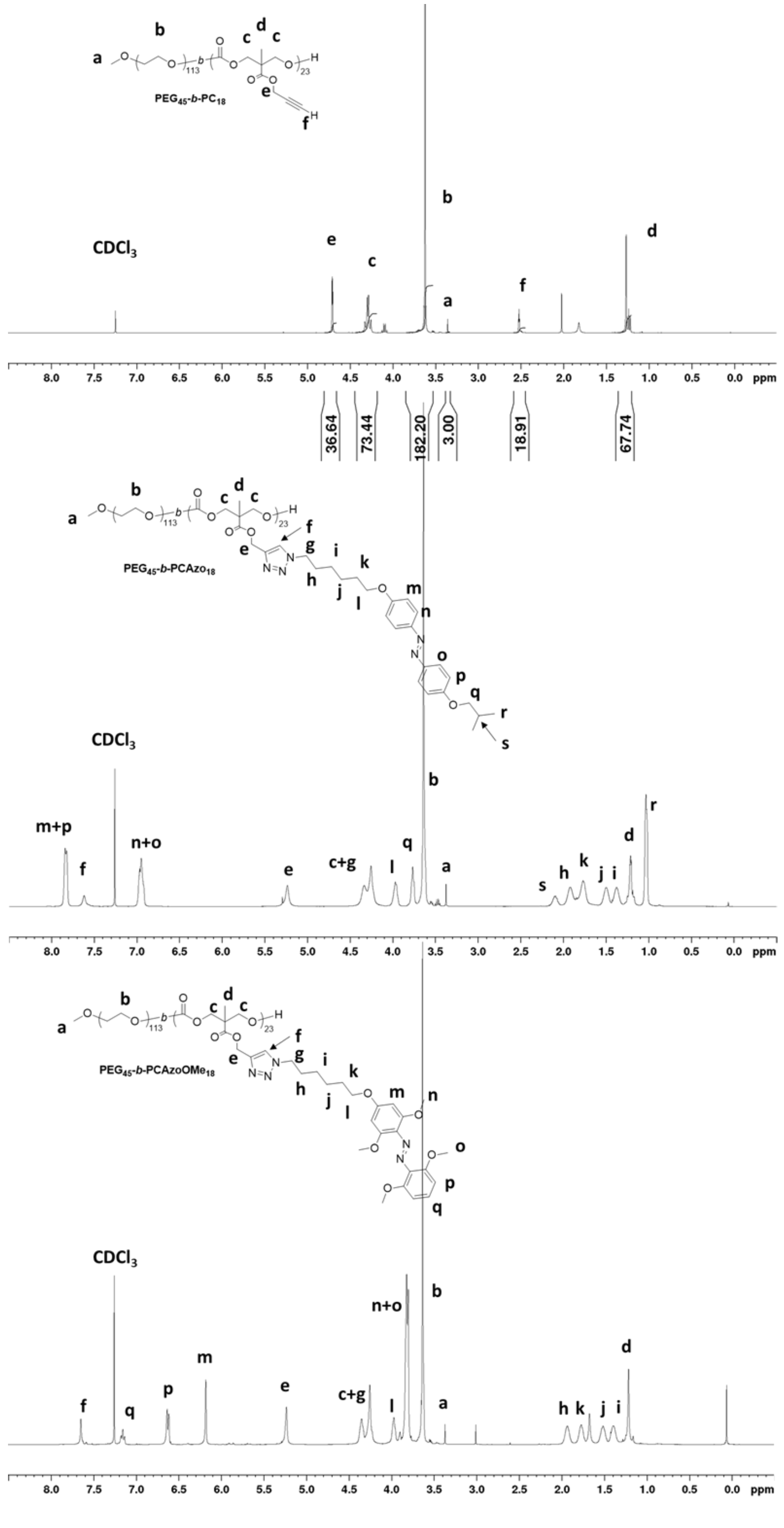

Figure S1. ^{1}H-NMR (400 MHz, $CDCl_3$) spectra of (from top to bottom) PEG_{45}-*b*-PC_{18}, PEG_{45}-*b*-$PCAzo_{18}$ and PEG_{45}-*b*-$PCAzoOMe_{18}$.

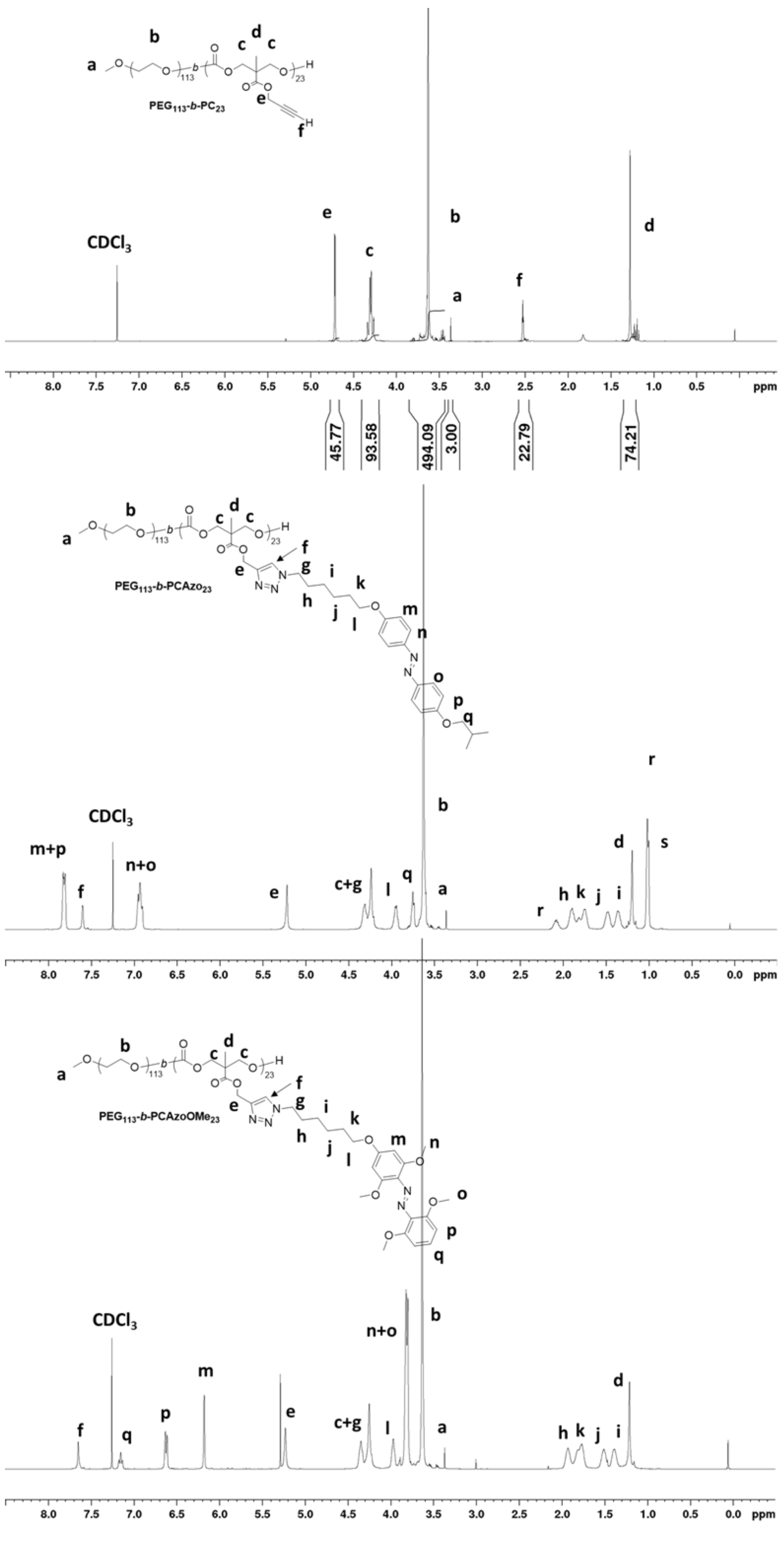

Figure S2. ^{1}H-NMR (400 MHz, $CDCl_3$) spectra of (from top to bottom) PEG_{113}-*b*-PC_{23}, PEG_{113}-*b*-$PCAzo_{23}$ and PEG_{113}-*b*-$PCAzoOMe_{23}$.

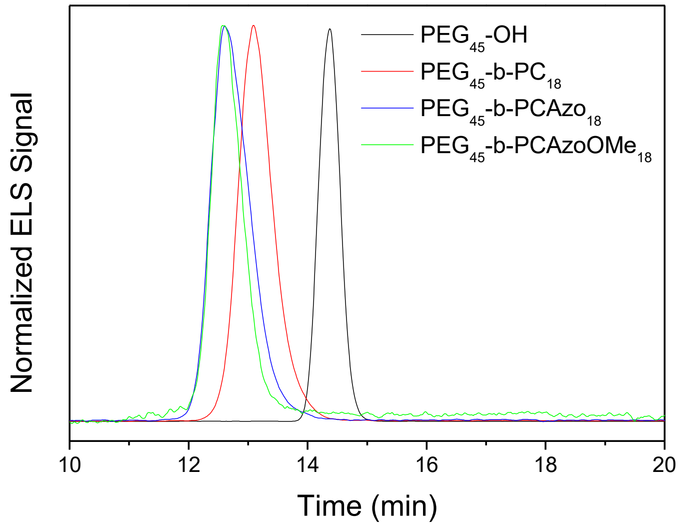

Figure S3. SEC traces for PEG$_{45}$-OH, PEG$_{45}$-*b*-PC$_{18}$, PEG$_{45}$-*b*-PCAzo$_{18}$ and PEG$_{45}$-*b*-PCAzoOMe$_{18}$.

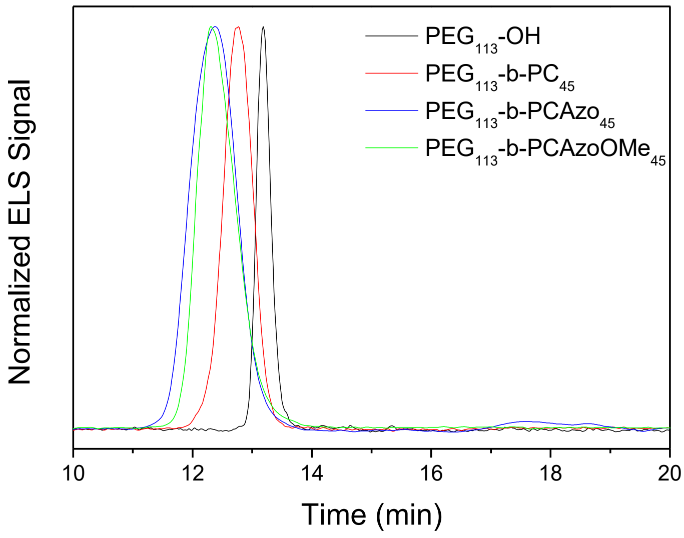

Figure S4. SEC traces for PEG$_{113}$-OH, PEG$_{113}$-*b*-PC$_{23}$, PEG$_{113}$-*b*-PCAzo$_{23}$ and PEG$_{113}$-*b*-PCAzoOMe$_{23}$.

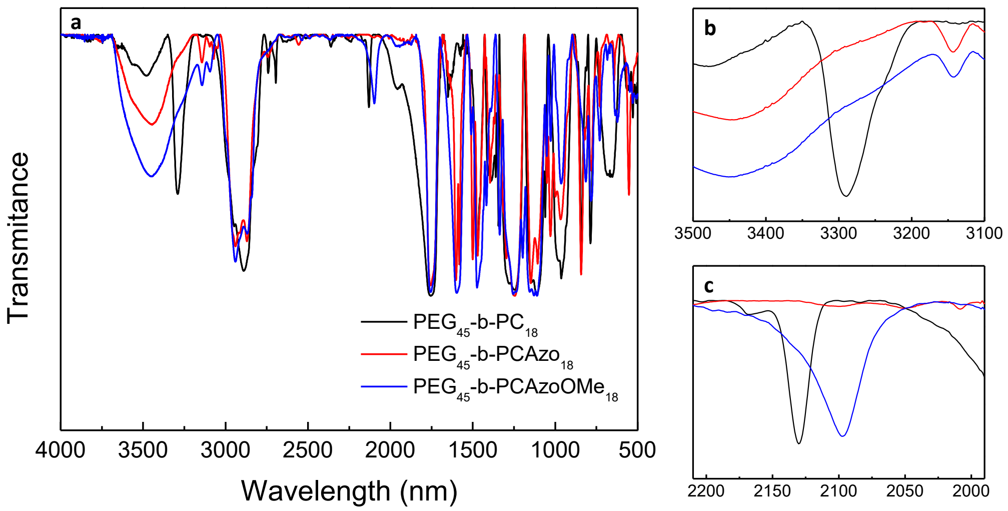

Figure S5. PEG45-*b*-PC18, PEG45-*b*-PCAzo18 and PEG45-*b*-PCAzoOMe18 FTIR spectrum (KBr disk) (a), and zoom to Csp-H zone (b) and Csp-Csp zone (c).

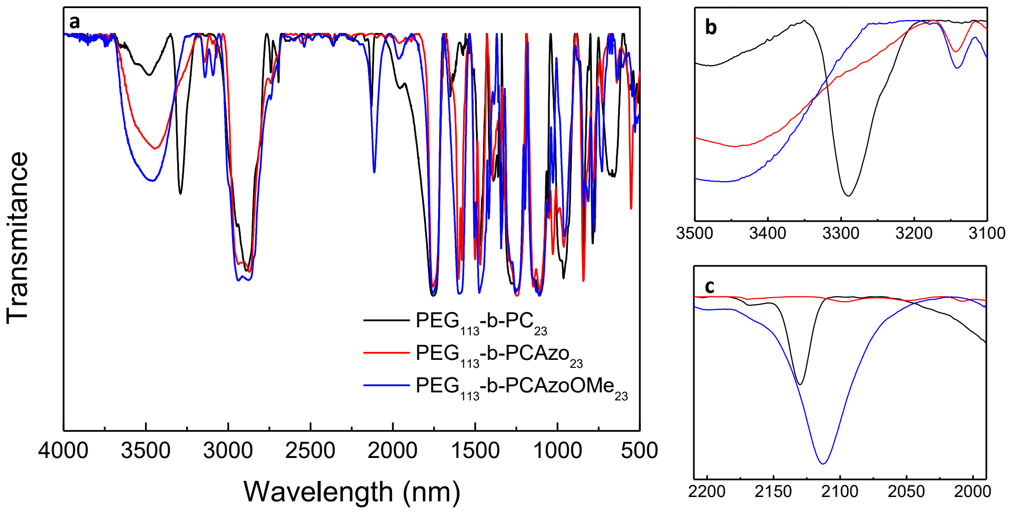

Figure S6: PEG113-*b*-PC23, PEG113-*b*-PCAzo23 and PEG113-*b*-PCAzoOMe23 FTIR spectrum (KBr disk) (a), and zoom to Csp-H zone (b) and Csp-Csp zone (c).

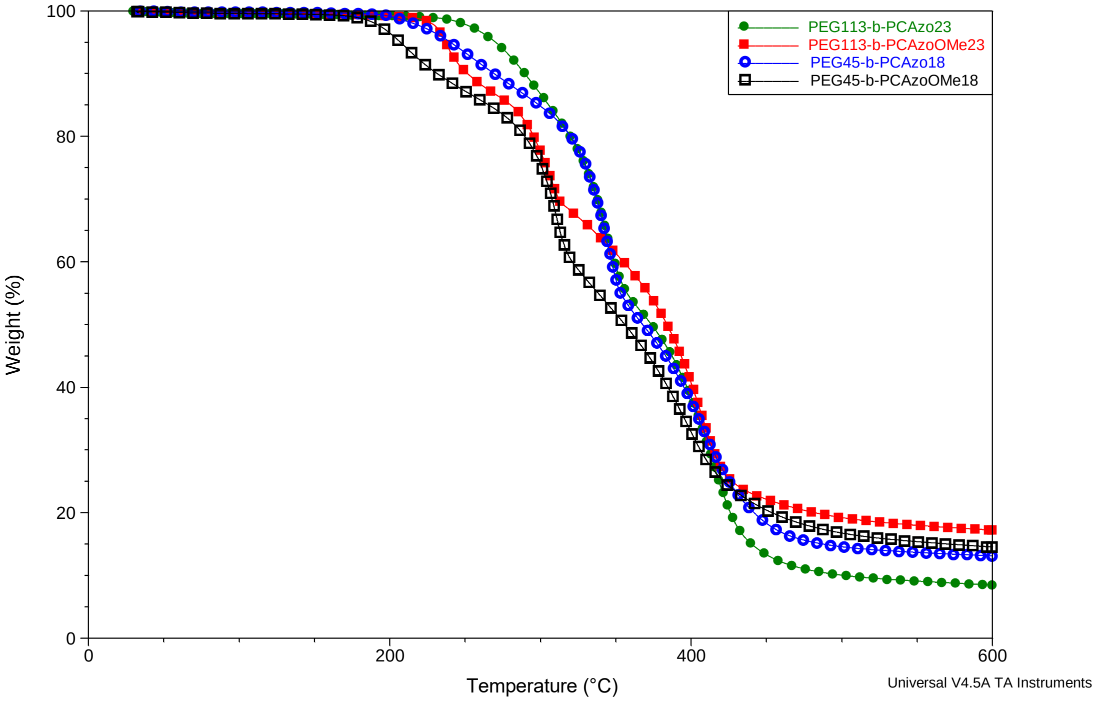

Figure S7. TGA curves registered at 10 °C min^{-1} heating rate under nitrogen atmosphere.

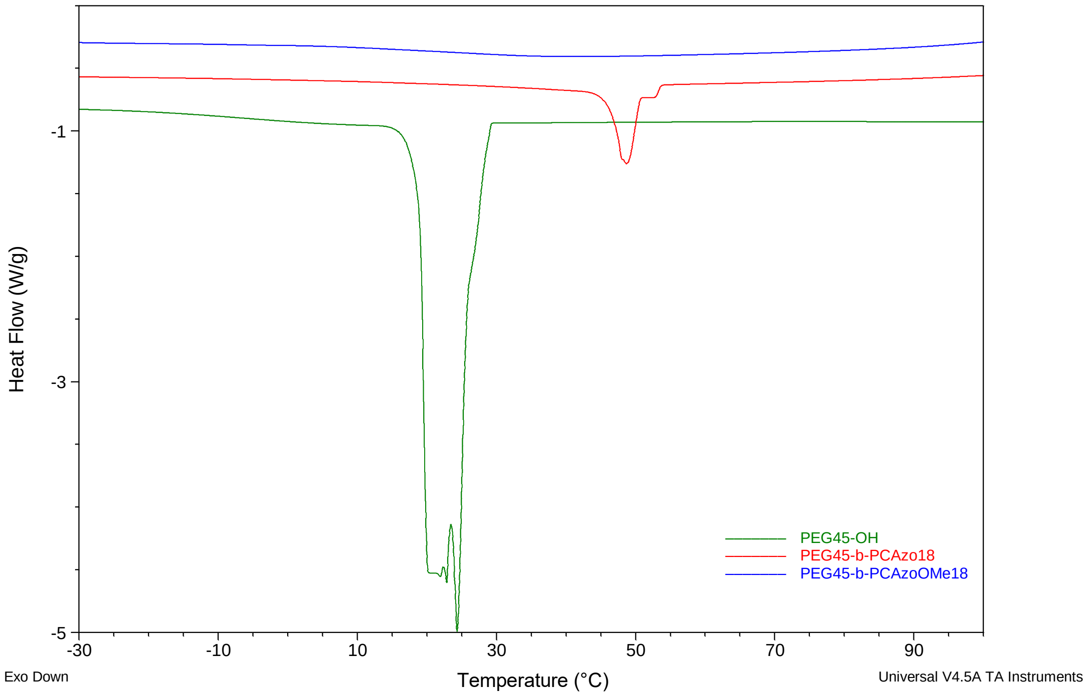

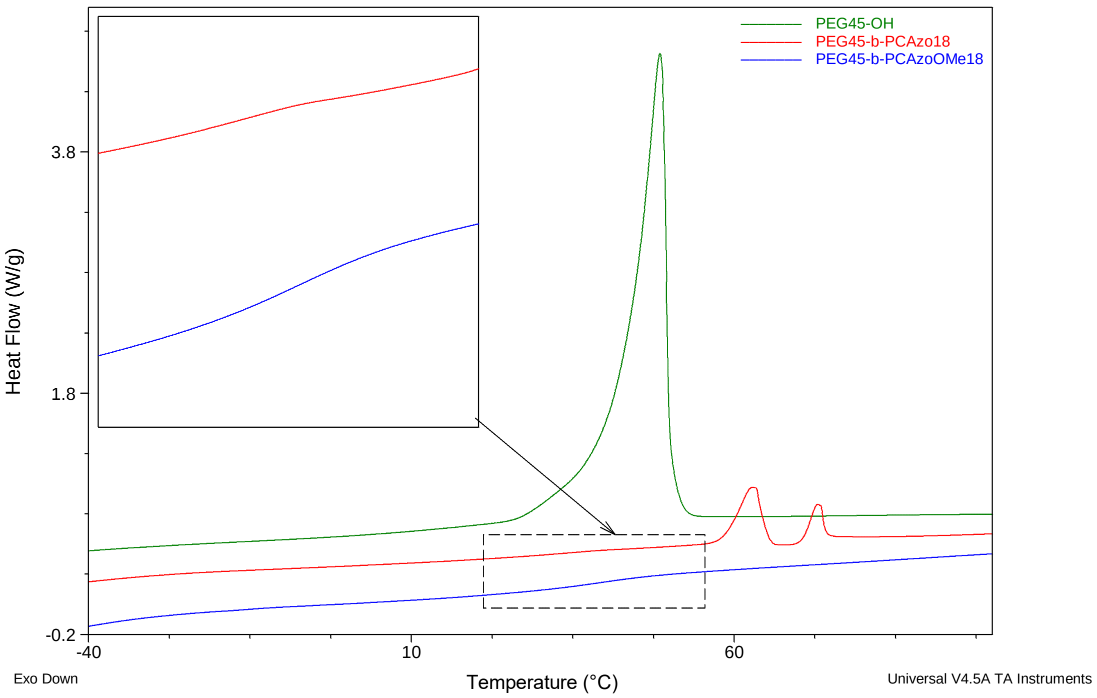

Figure S8. DSC curves registered on cooling (above) and subsequent heating (below) at a 10 °C min^{-1} scanning rate of PEG_{45}-OH, PEG_{45}-*b*-$PCAzo_{18}$ and PEG_{45}-*b*-$PCAzoOMe_{18}$.

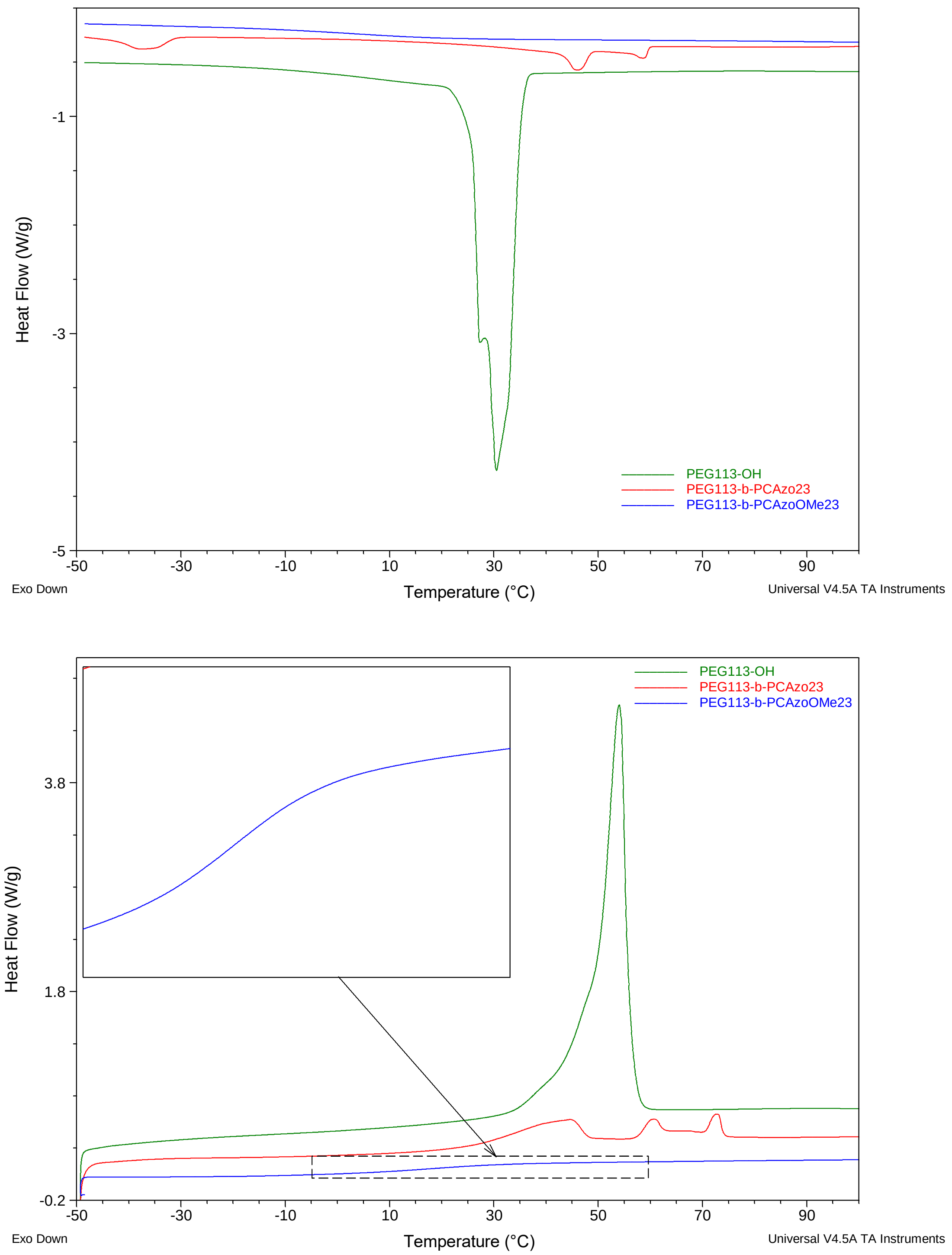

Figure S9. DSC curves registered on cooling (above) and subsequent heating (below) at a 10 °C min^{-1} scanning rate of PEG_{113}-OH, PEG_{113}-*b*-$PCAzo_{23}$ and PEG_{113}-*b*-$PCAzoOMe_{23}$.

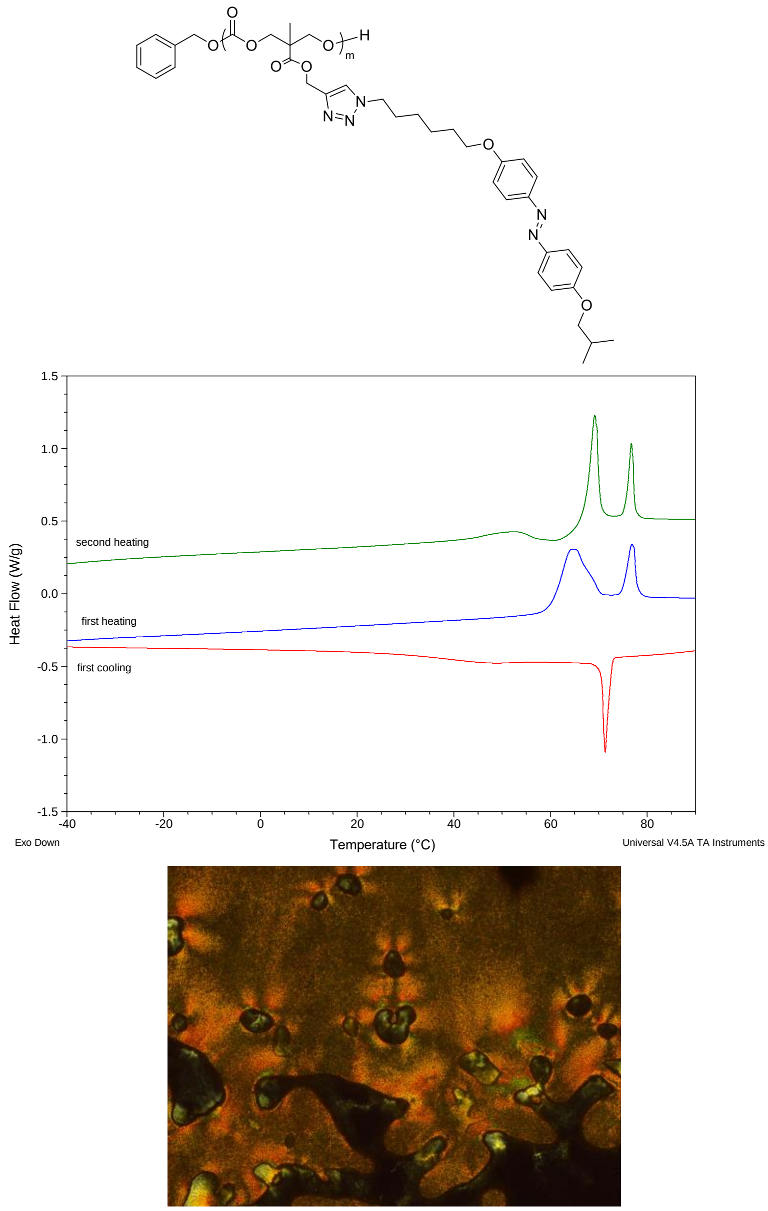

Figure S10. Structure of PCAzo (above), DSC curves registered at a 10 °C min^{-1} scanning rate (middle) and POM image (below) captured at 73 °C for homopolymer PCAzo. From the DSC scans, it was deduced that the isotropic liquid the mesophase vitrifies on cooling. On subsequent heating, the glass transition was measured at 46 °C followed by a cold crystallization process. Two endothermic transitions at 69 °C (ΔH = 5.4 kJ per mole of repeating unit) and 77 °C (ΔH = 2.5 kJ per mole of repeating unit) were registered corresponding to melting of the crystalline fraction and to the mesophase-to-isotropic liquid transition, respectively. No clearly identifiable textures were observed even after prolonged thermal annealing of the sample.

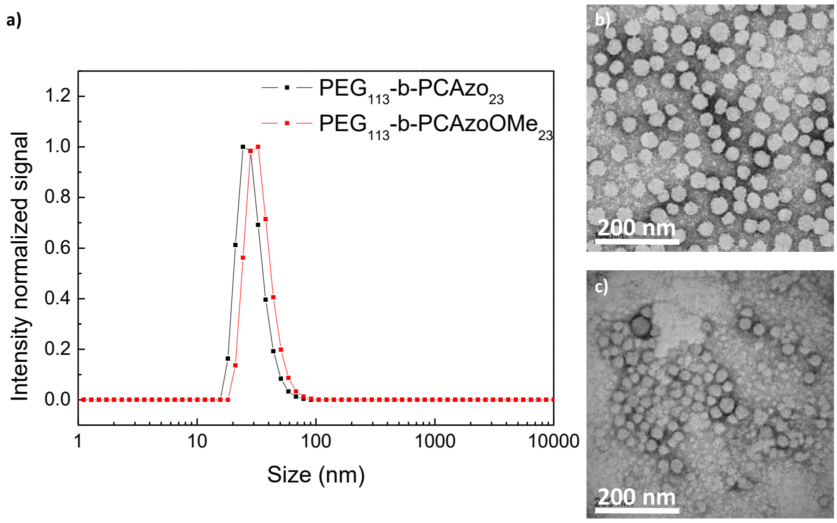

Figure S11. Analysis of the self-assembled structures of PEG113 amphiphilic block copolymers series. (a) DLS distribution curves of PEG113-*b*-PCAzo23 and PEG113-*b*-PCAzoOMe23. TEM image of (b) PEG113-*b*-PCAzo23 and(c) PEG113-*b*-PCAzoOMe23

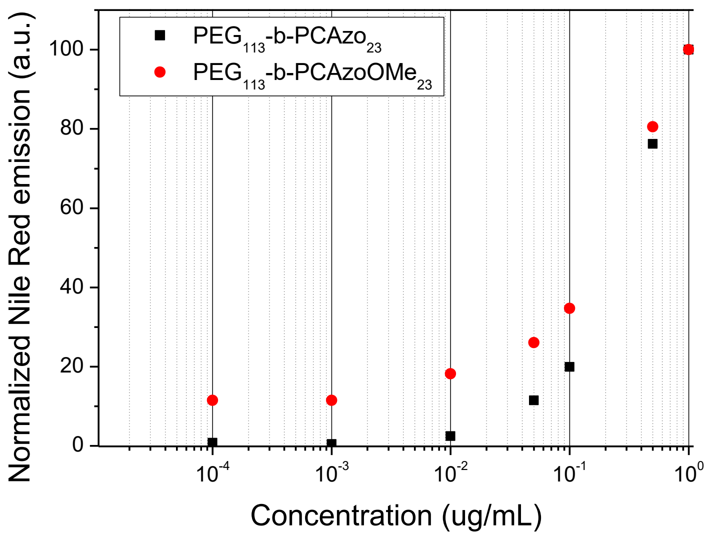

Figure S12. Normalized fluorescence emission of Nile Red at 606 nm (λexc = 550 nm) versus the PEG113-*b*-PCAzo23 and PEG113-*b*-PCAzoOMe23 concentration. CAC was determined from the intersection of the two extrapolated lines.

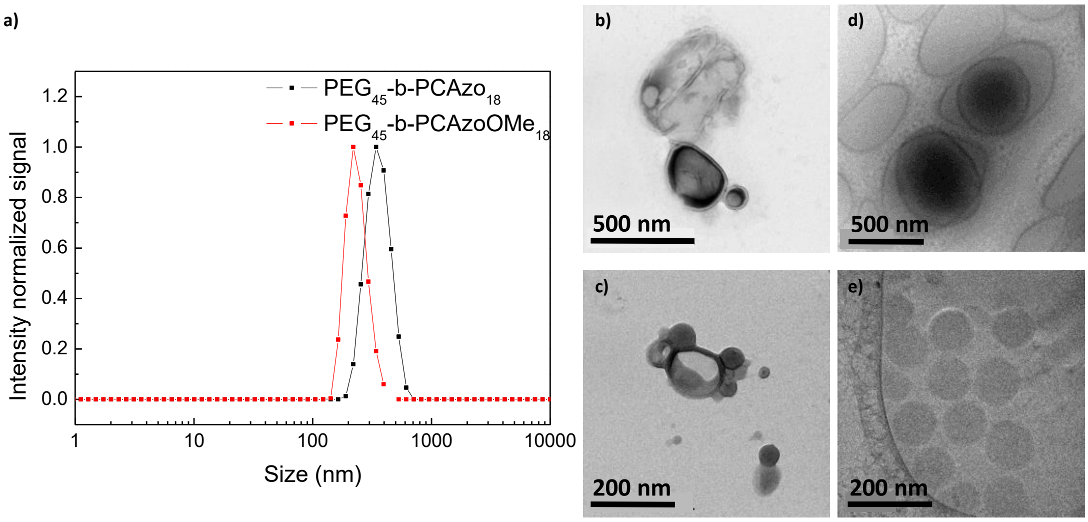

Figure S13. Analysis of the self-assembled structures of PEG_{45} amphiphilic block copolymers series. (a) DLS distribution curves of PEG_{45}-*b*-$PCAzo_{18}$ and PEG_{45}-*b*-$PCAzoOMe_{18}$. TEM images of (b) PEG_{45}-*b*-$PCAzo_{18}$ and (c) PEG_{45}-*b*-$PCAzoOMe_{18}$. Cryo-TEM image of (d) PEG_{45}-*b*-$PCAzo_{18}$ and (e) PEG_{45}-*b*-$PCAzoOMe_{18}$

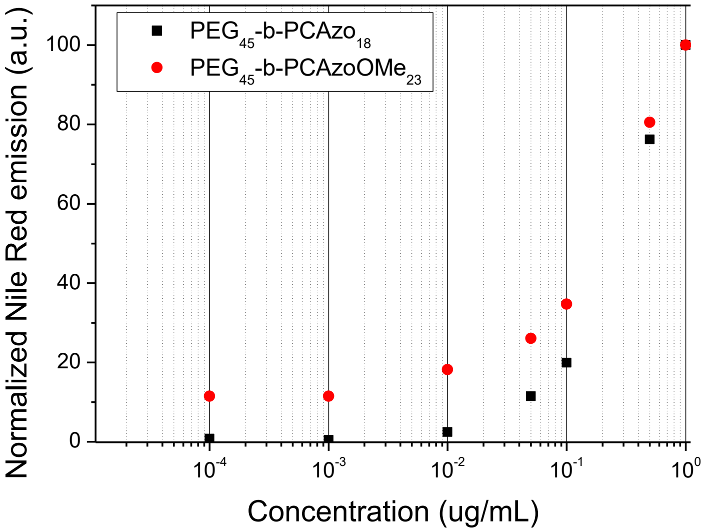

Figure S14. Normalized fluorescence emission of Nile Red at 606 nm (λexc = 550 nm) versus the PEG_{45}-*b*-$PCAzo_{18}$ and PEG_{45}-*b*-$PCAzoOMe_{18}$ concentration. CAC was determined from the intersection of the two extrapolated lines.

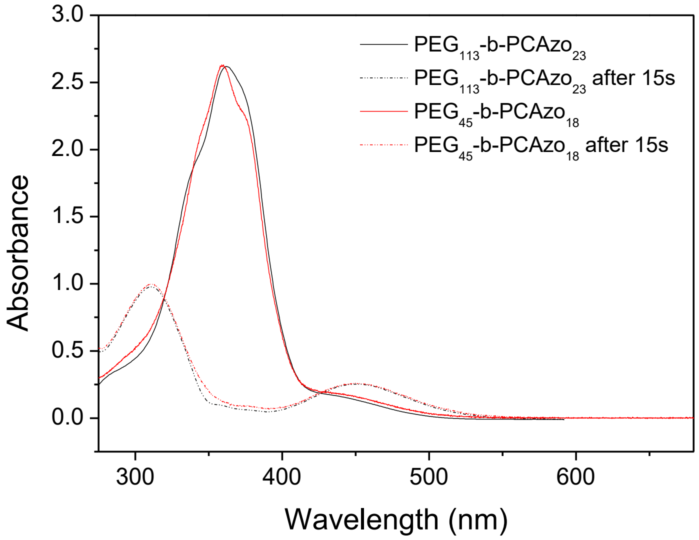

Figure S15. UV-Vis spectra of a 10^{-4} M (referred to the repetitive azobenzene unit) PEG_{113}-*b*-$PCAzo_{23}$ and PEG_{45}-*b*-$PCAzo_{18}$ solution in THF, before and after 15 s UV illumination.

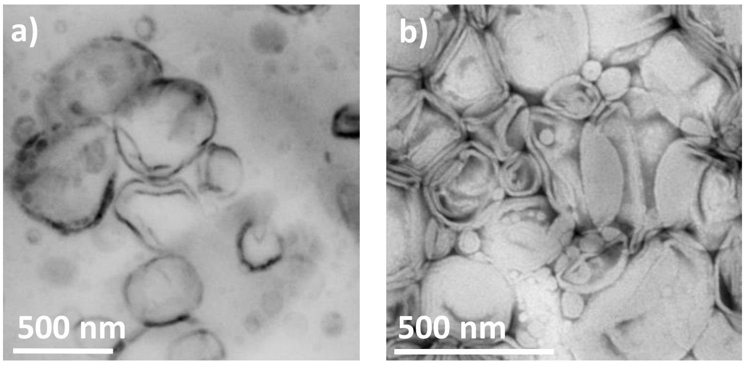

Figure S16. TEM images of PEG_{45}-*b*-$PCAzo_{18}$ self-assemblies before (a) and after 10 min low intensity UV light irradiation (b).

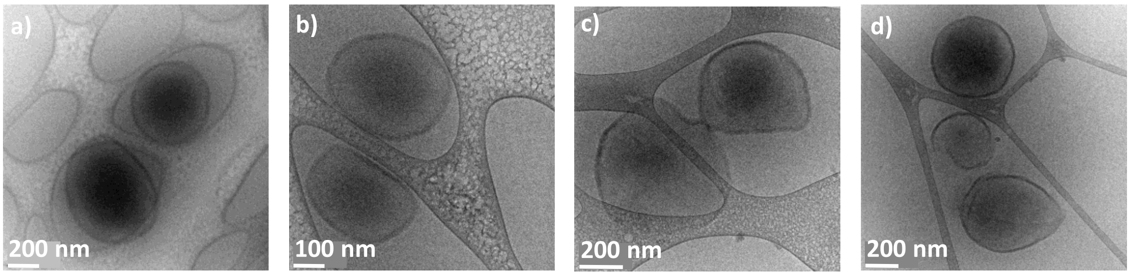

Figure S17. Cryo-TEM images of PEG_{45}-*b*-$PCAzo_{18}$ self-assemblies before (a and b) and after 10 min low intensity UV light irradiation (c and d).

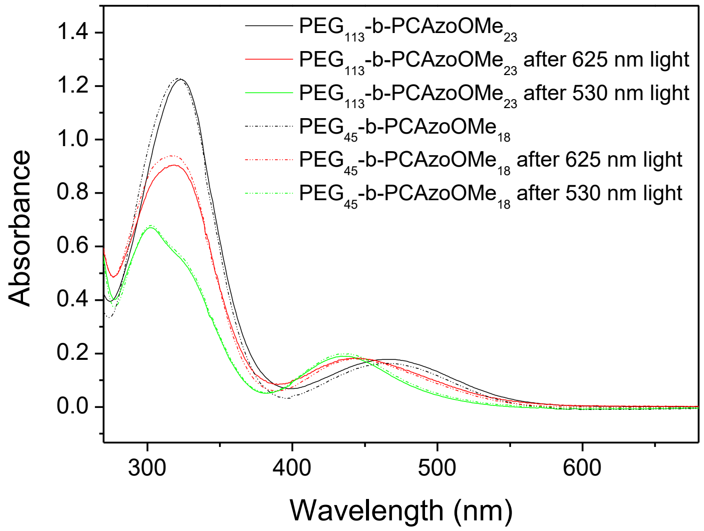

Figure S18. UV-Vis spectra of a 10^{-4} M (referred to the repetitive azobenzene unit) PEG_{113}-*b*-$PCAzoOMe_{23}$ and PEG_{45}-*b*-$PCAzoOMe_{18}$ solution in THF and photostationary state reached after 40 min under 625 nm light and after 30 s under 530 nm light.

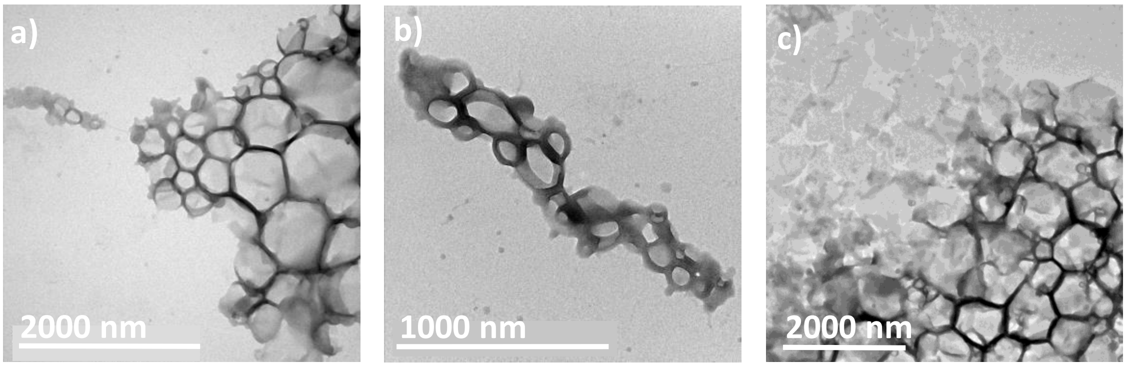

Figure S19. TEM images of PEG_{45}-*b*-$PCAzoOMe_{18}$ self-assemblies before (a), after 2 hours 625 nm light irradiation (b) and after 5 min 530 nm light irradiation (b).

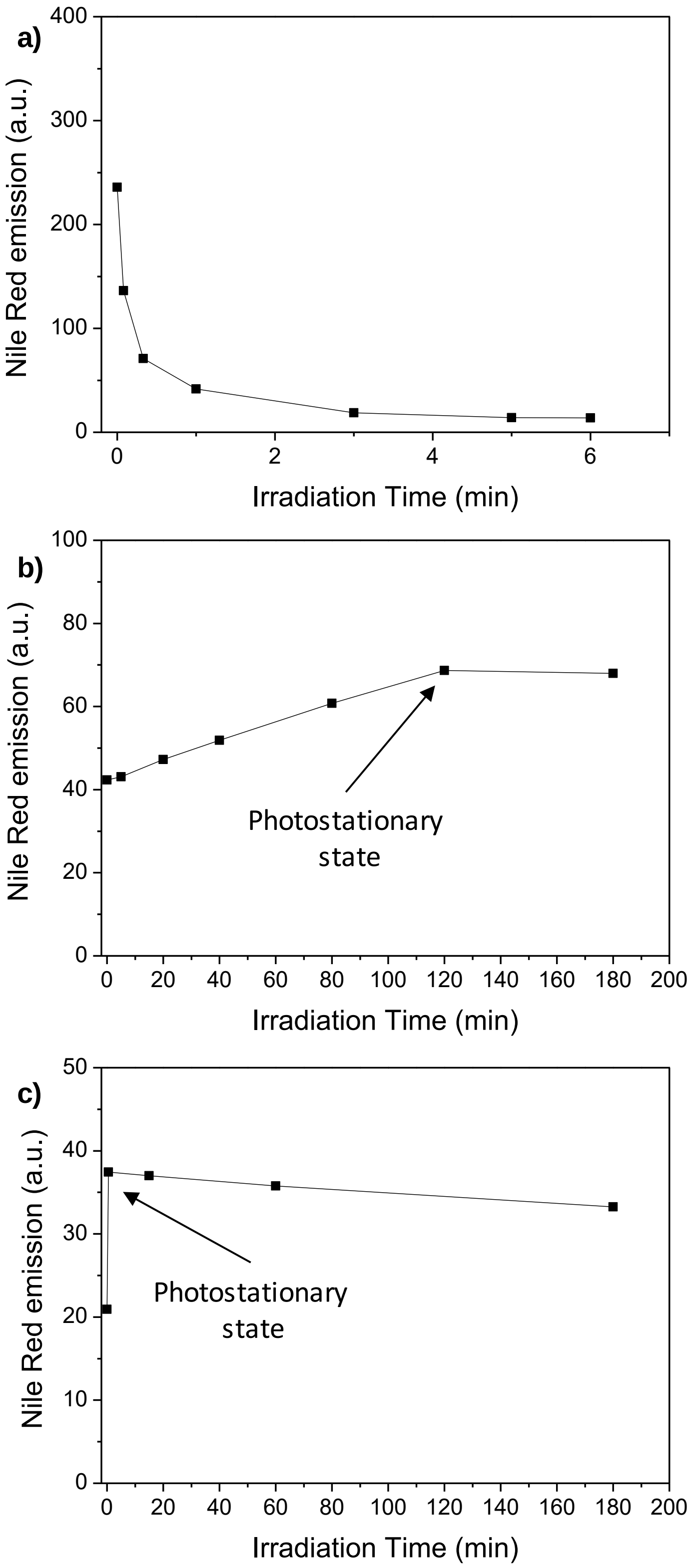

Figure S20. Cargo release profiles of the Nile Red loaded micelles for (a) PEG_{113}-*b*-$PCAzo_{23}$ micelles under UV light, (b) PEG_{113}-*b*-$PCAzoOMe_{23}$ micelles under 625 nm light and (c) PEG_{113}-*b*-$PCAzoOMe_{23}$ micelles under 530 nm.

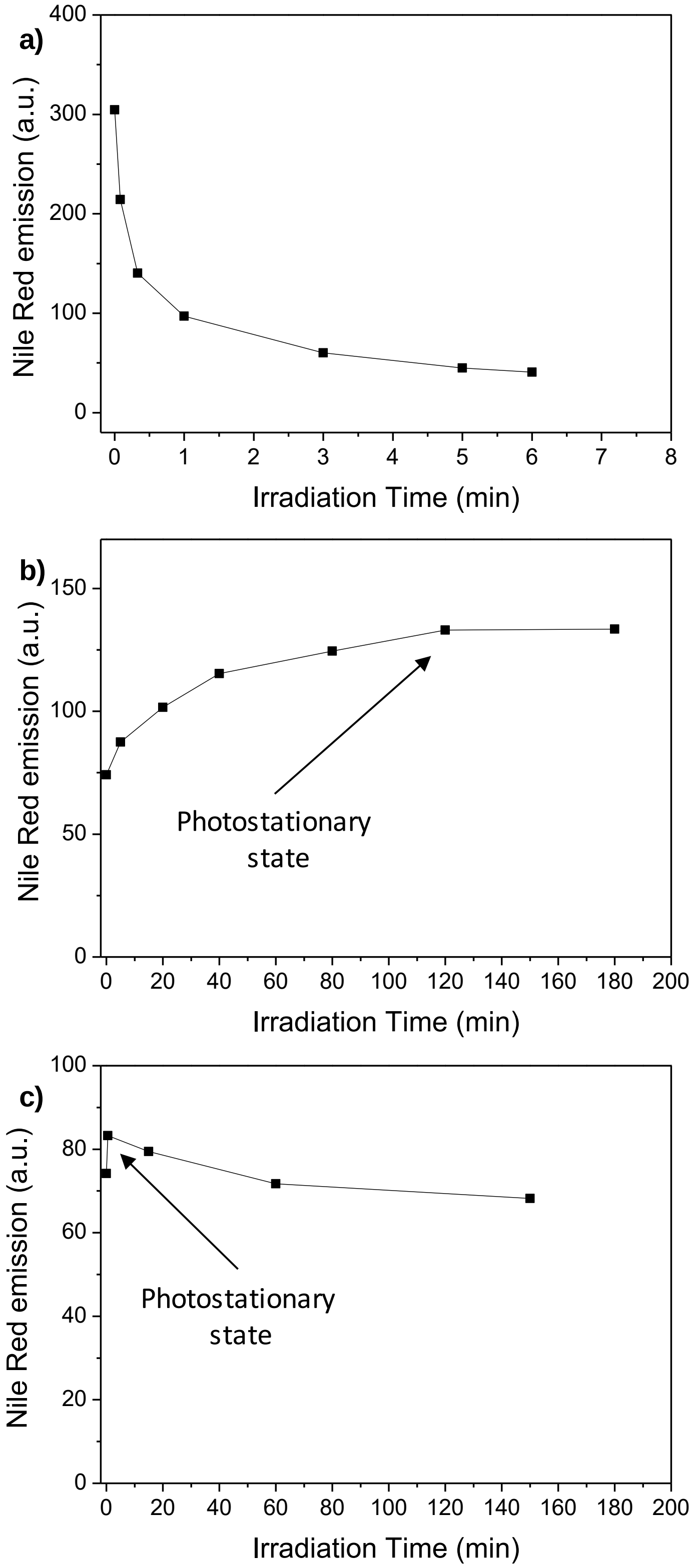

Figure S21. Cargo release profiles of the Nile Red loaded micelles for (a) PEG_{45}-*b*-$PCAzo_{18}$ vesicles under UV light, (b) PEG_{45}-*b*-$PCAzoOMe_{18}$ vesicles under 625 nm light and (c) PEG_{45}-*b*-$PCAzoOMe_{18}$ vesicles under 530 nm.

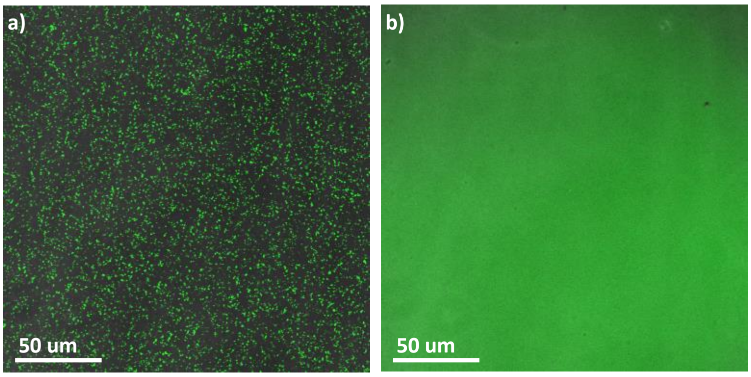

Figure S22. Fluorescence microscopy images of Rhodamine B loaded PEG_{45}-*b*-$PCAzo_{18}$ vesicles before (a) and after 10 min low intensity UV light irradiation (b).

Bibliography

1. Blasco, E.; Barrio, J. del; Sánchez-Somolinos, C.; Piñol, M.; Oriol, L. Light induced molecular release from vesicles based on amphiphilic linear-dendritic block copolymers. *Polym. Chem.* **2013**, *4*, 2246-2254.
2. Wang, G.; Yuan, D.; Yuan, T.; Dong, J.; Feng, N.; Han, G. A visible light responsive azobenzene-functionalized polymer: Synthesis, self-assembly, and photoresponsive properties. *Journal of Polymer Science Part A: Polymer Chemistry* **2015**, *53*, 2768–2775.
3. Blasco, E.; Barrio, J. del; Piñol, M.; Oriol, L.; Berges, C.; Sánchez, C.; Alcalá, R. Azobenzene-containing linear–dendritic block copolymers prepared by sequential ATRP and click chemistry. *Polymer* **2012**, *53*, 4604–4613.
